# PtNi-W/C with Atomically Dispersed Tungsten Sites Toward Boosted ORR in Proton Exchange Membrane Fuel Cell Devices

**DOI:** 10.1007/s40820-023-01102-9

**Published:** 2023-06-02

**Authors:** Huawei Wang, Jialong Gao, Changli Chen, Wei Zhao, Zihou Zhang, Dong Li, Ying Chen, Chenyue Wang, Cheng Zhu, Xiaoxing Ke, Jiajing Pei, Juncai Dong, Qi Chen, Haibo Jin, Maorong Chai, Yujing Li

**Affiliations:** 1 Beijing Key Laboratory of Construction Tailorable Advanced Functional Materials and Green Applications, School of Materials Science and Engineering, Beijing Institute of Technology, Beijing, 100081 People’s Republic of China; 2 State Power Investment Corporation Hydrogen Energy Company, Limited, Beijing, 102209 People’s Republic of China; 3 Faculty of Materials and Manufacturing, Beijing University of Technology, Beijing, 100124 People’s Republic of China; 4 Beijing Synchrotron Radiation Facility, Institute of High Energy Physics, Chinese Academy of Sciences, Beijing, 100049 People’s Republic of China

**Keywords:** Fuel cells, Membrane electrode assembly, PGM catalyst, Synergistic catalysis, Oxygen reduction

## Abstract

The performance of proton exchange membrane fuel cells is heavily dependent on the microstructure of electrode catalyst especially at low catalyst loadings. This work shows a hybrid electrocatalyst consisting of PtNi-W alloy nanocrystals loaded on carbon surface with atomically dispersed W sites by a two-step straightforward method. Single-atomic W can be found on the carbon surface, which can form protonic acid sites and establish an extended proton transport network at the catalyst surface. When implemented in membrane electrode assembly as cathode at ultra-low loading of 0.05 mg_Pt_ cm^−2^, the peak power density of the cell is enhanced by 64.4% compared to that with the commercial Pt/C catalyst. The theoretical calculation suggests that the single-atomic W possesses a favorable energetics toward the formation of *OOH whereby the intermediates can be efficiently converted and further reduced to water, revealing a interfacial cascade catalysis facilitated by the single-atomic W. This work highlights a novel functional hybrid electrocatalyst design from the atomic level that enables to solve the bottle-neck issues at device level.

**Supplementary Information:**

The online version contains supplementary material available at 10.1007/s40820-023-01102-9.

## Highlights

- A hybrid electrocatalyst consisting of PtNi-W alloy nanocrystals loaded on carbon surface with atomically dispersed W sites was realized.
- Single-atomic W formed protonic acid sites and established an extended proton transport network at the catalyst surface.
- Peak power density is enhanced by 64.4% compared to that with the commercial Pt/C catalyst in fuel cell as cathode at ultra-low loading of 0.05 mg_Pt_ cm^−2^.

**Supplementary Information:**

The online version contains supplementary material available at 10.1007/s40820-023-01102-9.

## Graphical abstract

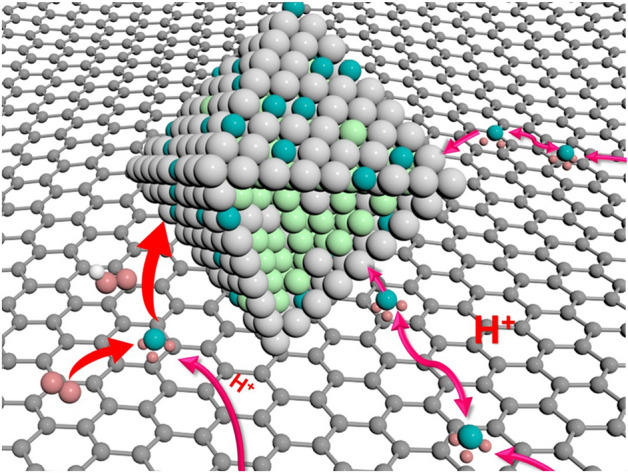

## Introduction

High-performance platinum-group-metal (PGM) with minimized loading and high adaptability is considered as the main technological challenge for the large-scale commercialization of the proton exchange membrane fuel cells (PEMFCs), which needs to be accomplished through the design of highly efficient electrocatalytic material and three-phase microenvironment [1, 2]. Specifically, for PEMFC systems implemented in underwater vehicle or space missions, the fuel cell will mostly be operated with feeding of hydrogen and pure oxygen [3, 4]. When oxygen is supplied to the cathode, the oxygen reduction reaction (ORR) rate will depend not only on the diffusion of oxygen molecules, but also on the micro-interface in the catalyst layer, which largely influences the transport of protons on the cathode side. In membrane electrode assembly (MEA) devices, the electrode holds a porous structure formed by the catalyst and solid electrolyte, wherein the electrolyte network facilitates proton transport throughout the catalytic layer [5, 6]. However, as catalytic reactions can only proceed at regions rich of three-phase micro-interface structure, the performances of the MEA show strong dependence on the catalyst ink formulation and processing technique, leading to the distinct microstructure and hence the drastically varied performances [7, 8]. Therefore, in addition to ionomer electrolyte, the proton transport should be facilitated by further means in the design of catalyst layer for ensuring the catalytic reaction. In the scenarios with ultra-low PGM loading, the development of a highly efficient proton-transport microstructure should be of higher importance, and also a great challenge for the research and development of MEA.

Intensive efforts have been carried out to develop highly efficient PGM catalyst for MEA [9-11]. Some emerging catalytic materials have been reported to enhance the performance in devices involving novel mechanisms, such as the hybrid catalytic site strategy. By combining transition metal single-atom catalytic (SAC) sites, such as Fe [12, 13], Co [14, 15], Cu and Zn SACs [16, 17], with the PGM-based nanoparticles, the synergistic effects have been reported to enhance the catalytic activity and stability. A typical work on ORR electrocatalyst loaded core–shell Pt alloy nanoparticles on single-atom Fe catalyst reported by Liu et al*.* [18]. The unique microstructure, together with the interaction between the Pt alloy and the single-atom Fe sites, boosted the MEA performance. To achieve a microstructure with higher power density, the supporting carbon-based materials with nitrogen- or oxygen-rich moieties have been reported, wherein the enhancements are considered to be associated with the improved oxygen mass transfer in the catalyst layer [19, 20]. Nonetheless, very few strategies have been developed to improve the proton transport in MEA device. Challenges remain in the design of controllable microstructure with improved proton transport in the catalytic layer especially in the pure oxygen operating condition.

The hydrogen spillover from the Pt to the support (tungsten oxide, WO_3_) has been discovered in 1960s [21, 22]. More recently, the WO_3_ implemented in anode has been demonstrated by Kim et al*.*, to facilitate the durability of MEA in cyclic start-up/shut-down operations through a metal–insulator transition mechanism. The WO_3_ lattice can switch between metal and insulator by proton intercalation and deintercalation, whereby the corrosion of the cathode induced by the oxygen leakage in the anode can be mitigated [23]. Additionally, Vlachos et al. demonstrated the high catalytic activity of the Pt-WO_x_ by modulating the dynamics of atomic Brønsted acid site originating from hydrogen spillover on WO_x_ [24]. However, there are few reports of hydrogen overflow response under oxygen conditions on the cathode side of the cell [25-27].

Herein, we report a hybrid electrocatalytic structure consisting of carbon-based single-atomic tungsten (W) as a supporting material loaded with PtNi-W nanoparticles (denoted as PtNi-W/C). The as-designed catalyst material with hybrid active sites resulted in an increase in mass activity (MA) compared with the commercial Pt/C catalyst. When implemented as cathode in MEA at ultra-low PGM loading of 0.05 mg_Pt_ cm^−2^, the synergistic effect owing to the efficient proton transport and oxygen reduction kinetics enables a 64.4% increase in power density compared with the MEA fabricated with commercial catalysts, by displaying a peak power density at 2030 mW cm^−2^ in H_2_-O_2_ operation condition.

## Experimental Methods

### Chemicals and Materials

Platinum acetylacetonate (Pt(acac)_2_, 97%), nickel acetylacetonate (Ni(acac)_2_, 95%), tungsten hexacarbonyl (W(CO)_6_, 99.9%), ascorbic acid (C_6_H_8_O_6_, 99%), benzoic acid (C_7_H_6_O_2_, 99.5%), N, N-Dimethylformamide (C_3_H_7_NO, 99.5%) and anhydrous ethanol (ETOH, 99.5%), were purchased from Aladdin. Vulcan XC-72R carbon black was received from Cabot Corporation. Acetone (C_3_H_6_O, 99.8%) was purchased from Beijing Tongguang Fine Chemicals Company, concentrated perchloric acid (HClO_4_, 70%) from Sigma-Aldrich, and commercial Pt/C (20 wt% Pt) from Johnson Matthey. Hydrogen (H_2_, 99.999%) is produced by a hydrogen generator. Pressurized air is prepared by compressing and filtering the ambient air. Carbon monoxide (CO, 99.999%), Oxygen (O_2_, 99.999%) and Nitrogen (N_2_, 99.999%) gases were obtained from Beijing Huatong Jingke Gas Chemicals Company. All chemicals were used as received without further purification.

### Synthesis of Transition Metal Doped Pt and Undoped Pt

Referring to the preparation protocol of PtNi/C in previous paper [28], Pt(acac)_2_ (0.40 g or 1.00 mmol) and Ni(acac)_2_ (0.17 g or 0.65 mmol) were first dispersed in C_3_H_6_O (20 mL) by sonication, and the carbon black was uniformly dispersed into the mixture by stirring. The dried mixture was then heated to 200 °C at 5 °C min^−1^ and maintained at 200 °C for 1 h in H_2_/CO (5/120 cm^3^ min^−1^). And N_2_ was used for purging the system before collecting the product. After that 50 mg PtNi/C with 10 mg W(CO)_6_, 20 mg C_6_H_8_O_6_, 10 mg C_7_H_6_O_2_ were dispersed in DMF (20 mL) and held at 160 °C for 12 h. The PtNi-W/C catalyst was washed three times by centrifugation using a mixture of ethanol and acetone. Finally, the catalyst was dried in an oven at 60 °C for later use.

### Characterization

X-ray powder diffraction (XRD) patterns were recorded on a Bruker D8 Focus XRD at a scan rate of 5° min^−1^_._ The wide-angle x-ray scattering (WAXS) data were obtained at BL14B1 of the Shanghai Synchrotron Radiation Facility (SSRF) using X-ray with a wavelength of 0.6887 Å [29]. Transmission electron microscopy (TEM) images were recorded on a JEOL JEM-2100. HR-TEM images and HAADF-STEM images were obtained on a FEI Titan G2 microscope equipped with an aberration corrector for probe-forming lens and a Bruker Super-X EDS detector operated at 300 kV. The size distribution of the nanocrystals was carried out by randomly measuring 100 nanocrystals in different regions. X-ray photoelectron spectroscopy (XPS) measurement was taken on Thermo ESCALAB 250Xi. The metal contents in the catalysts were measured by inductively coupled plasma-atomic emission spectrometer (Teledyne Leeman Labs Prodigy7). The thickness images of RDE catalyst films were recorded on a BJMICRO optical microscope.

### Electrochemical Properties of Catalysts

All the electrochemical measurements were taken in a three-electrode test cell and recorded at room temperature (25 °C) with CHI 760E electrochemical workstation. The saturated calomel electrode (SCE) was used as reference electrode and platinum (Pt) plate as a counter electrode. The calibration of the SCE is carried out according to a well-established protocol from the literature [30]. The ink was made with ultrapure water (200 µL), isopropanol (IPA, 200 µL), and Nafion solution (4 µL, 5 wt%). The loading of precious metal (Pt) on glassy carbon electrode was fixed at 20 µg cm^−2^. For all the ORR measurements, the 0.1 M HClO_4_ electrolyte was purged with oxygen prior to and during the measurement to make sure the electrolyte was saturated with O_2_. The cyclic voltammetric curves were recorded from 0.1 to 1.1 V at a sweep rate of 50 mV s^−1^. And electrochemically active surface area (ECSA) is used to estimate intrinsic ORR activities in the RDE measurement. The polarization curve was swept from 0.1 to 1.1 V at a sweep rate of 20 mV s^−1^ at a rotating rate of 1,600 rpm. The ADT was carried out by repeatedly polarizing the electrode between 0.6 and 1.0 V at the scan rate of 50 mV s^−1^ in oxygen-saturated 0.1 M HClO_4_ electrolyte using graphite as counter electrode [31]. The currents were corrected by background subtraction and iR correction. The limiting diffusion current *J*_d_ and the measured current *J* @0.9 V for the catalyst in the ORR catalytic reaction can be obtained from the LSV test results, and a calculation based on the Koutecky–Levich equation can be performed to find the value of the kinetic current *J*_k_ for the catalyst, using Eq. (1):

1 $$\frac{1}{J} = \frac{1}{{J_{{\text{k}}} }} + \frac{1}{{J_{{\text{d}}} }}$$

The TOF is calculated by Eq. (2):

2 $$TOF = \frac{{N_{A} \times j_{m} }}{{SD_{mass} \times F}}$$

where *j*_m_ represents the kinetic mass-specific activity measured from electrochemical measurement, the site density per gram of catalyst is known as SD_mass_ and can be calculated using Eq. (3):

3 $$SD_{{{\text{mass}}}} = \frac{SD \times ECSA}{{{\text{m}}_{catalyst} }}$$

And the active site density (SD) was followed the relation as described by Eq. (4):

4 $$SD = \frac{{Q_{tip} \div F \times N_{A} }}{ECSA}$$

where the electric quantity at tip (*Q*_tip_) was derived by background subtraction and integration of I.

### DFT Calculations

All geometric optimization, transition state finding and Gibbs free energy calculation are based on density functional theory (DFT), using the Vienna ab initio simulation package (VASP) and VASP Transition State Tools (VTST) [32-35]. The interaction between valence electrons and nuclei can be easily simulated by the Projector-augmented wave (PAW) potential database [36, 37]. The Perdew–Burke–Ernzerhof (PBE) and Generalized-Gradient Approximation (GGA) methods of exchanging correlation functions can be used to investigate electron transfer and correlation well [38]. The plane wave extended energy cutoff is 450 eV, and the first Brillouin zone is sampled with high precision on a 2 × 2 × 1 Monkhorst–Pack k-point grid based on a free energy error test. A Gaussian coverage width of 0.1 eV is set to calculate the structural relaxation and energy [39]. The force of the unconstrained atom of the conjugate gradient algorithm is set to be less than 0.01 eV Å^−1^ and the energy convergence value is 1 × 10^−5^ eV. The saddle point and the minimum energy path are found using the climbing image nudged elastic band (CI-NEB) method when the convergence value of the force is set to 0.03 eV Å^−1^ [40, 41]. For a better understanding of the synergistic interaction between W_SA_O_3_ and PtNi-W, hydrogen binding energy (HBE) calculations were performed in acidic media [42].

In acidic medium, the complete 4e^−^ ORR occurs via the following steps [15, 43, 44]:

5 $${4}({\text{H}}^{ + } + {\text{e}}^{ - } ) + {\text{O}}_{2} \to {3}({\text{H}}^{ + } + {\text{e}}^{ - } ) \, + *{\text{OOH}}$$

6 $${3}({\text{H}}^{ + } + {\text{e}}^{ - } ) + *{\text{OOH}} \to {2}({\text{H}}^{ + } + {\text{e}}^{ - } ) + *{\text{O}} + {\text{H}}_{2} {\text{O}}$$

7 $${2}({\text{H}}^{ + } + {\text{e}}^{ - } ) + *{\text{O}} + {\text{H}}_{2} {\text{O}} \to ({\text{H}}^{ + } + {\text{e}}^{ - } ) + *{\text{OH}} + {\text{H}}_{2} {\text{O}}$$

8 $$({\text{H}}^{ + } + {\text{e}}^{ - } ) + *{\text{OH}} + {\text{H}}_{2} {\text{O}} \to {\text{2H}}_{2} {\text{O}}{.}$$

### MEA Fabrication and Fuel Cell Test

The prepared PtNi-W/C and Pt/C (20 wt%, Johnson Matthey) catalysts were used as the cathode catalyst, and the Pt/C (60 wt%, Johnson Matthey) as the anode catalyst. The catalyst was dispersed in water/ isopropyl alcohol with DuPont D2020® perfluorosulfonic acid (PFSA) ionomer. After that the desired catalyst ink was prepared by ultrasonication. The ionomer/carbon ratio is controlled at 0.9, and the water/alcohol volume ratio at 1.0. Use an airbrush to evenly spray the ink on both sides of the Nafion membrane (Gore, M820.15). After drying, the CCM and the gas diffusion layer (GDL, Sigracet 29 BC) are heated at 130 °C and 0.14 MPa for 2 min to assemble the MEA. Apply a torque of 4 N·m to assemble the MEA and the fixture into a standard 5 cm^2^ cell. Fixed cathode and anode catalyst loadings are used for quantitative activity comparisons. A loading of 0.05 mg_Pt_ cm^−2^ was used on the cathode side to compare the activity and 0.10 mg_Pt_ cm^−2^ loading to explore the degradation of durability. The anode side of all MEA adopts the same catalyst loading as commercial MEA, 0.10 mg_Pt_ cm^−2^. Both the anode and cathode loadings were confirmed by ICP-AES measurements.

After connecting the standard 5 cm^2^ cell to the Hephas 330 fuel cell test system, the cell was activated by high current, and the polarization curve tests under the conditions of H_2_-O_2_ and H_2_–Air were carried out in sequence. The polarization tests were performed at a fixed gas flow rate (anode: 1500 mL min^−1^; cathode: 2000 mL min^−1^) to reduce the influence of concentration polarization. The test conditions used were 80 °C, 100% humidity, and an absolute back pressure setting of 150 kPa (equivalent to air pressure of 1 bar). Keeping the environmental conditions of the polarization test unchanged, the Gamry Interface 5000E electrochemical workstation was selected for electrochemical impedance measurement. The test with a frequency range of 100 mHz to 1 MHz was performed under the DC current set to 2.5 A and 10% AC current disturbance. According to the catalyst durability test protocol recommended by the US Department of Energy, electrocatalyst durability test was conducted for 10 k cycles with the cell's anode set to 200 mL min^−1^ of hydrogen and the cathode set to 75 mL min^−1^ of nitrogen. For the fuel cell testing, the geometric surface area is used to calculate MEA power density and current density.

## Results and Discussion

### Structure and Morphology Characterization

**Fig. 1:**
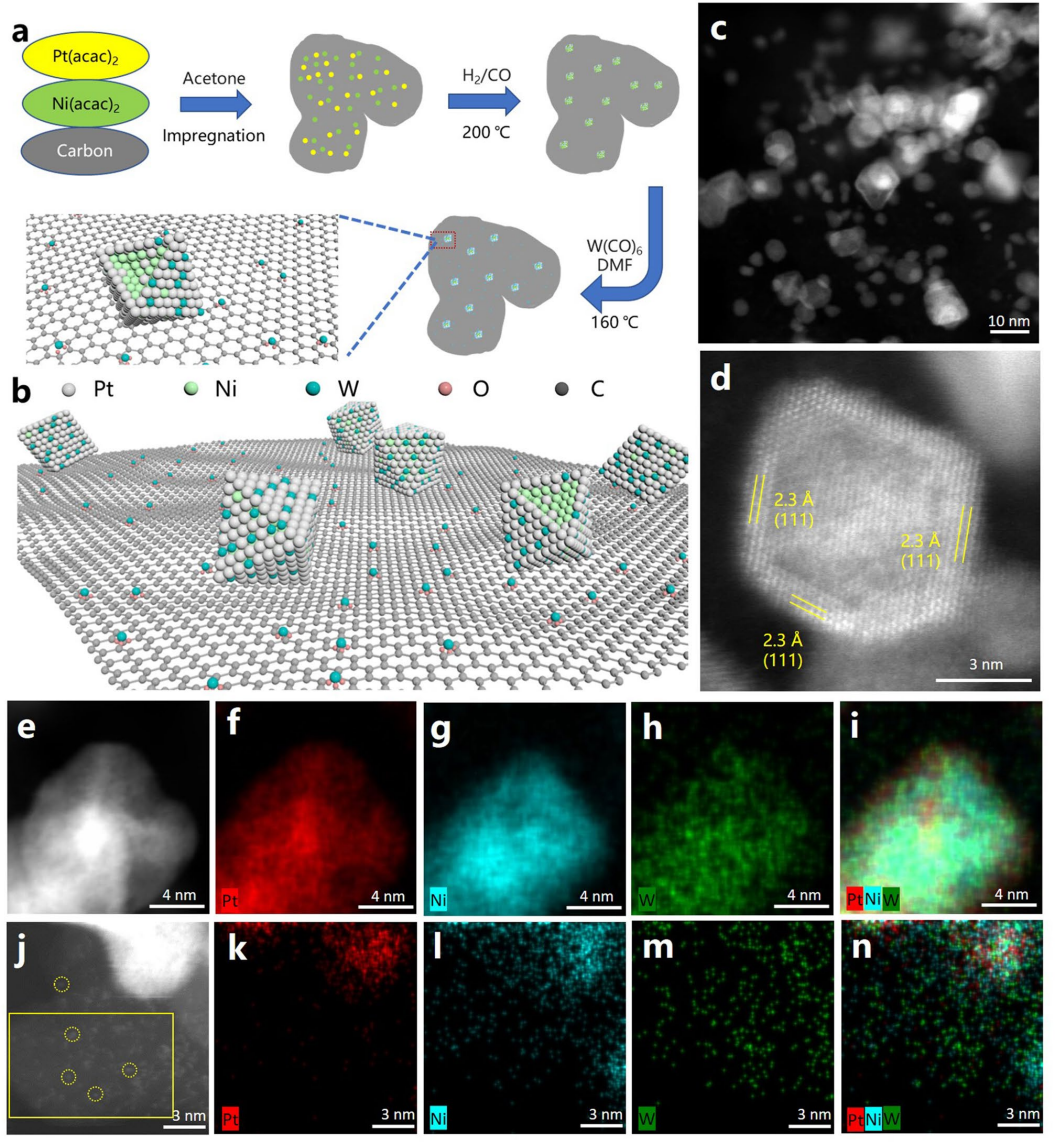
**a** Schematic illustration of the synthetic protocol and **b** nanostructure of PtNi-W/C. **c**, **d** HAADF-STEM images of PtNi-W/C catalyst. **e**-**n** HAADF-STEM images with elemental mapping of **e**-**i** a single PtNi-W nanoparticle and **j**-**n** the carbon support (**j** highlight the region selected for EDX spectrum and mapping in SI (Fig. S3a) with a yellow rectangle), where the Pt, Ni, W, and mixed Pt/Ni/W distributions are displayed

We develop a two-step method combining vapor phase reduction followed with wet-chemical treatment to synthesize the hybrid catalyst. Upon the formation of PGM alloy catalyst (denoted as PtNi/C) through an impregnation method using low-temperature vapor phase reduction in mixed gas of carbon monoxide and hydrogen (Fig. 1a), the powder is then treated in dimethylformamide (DMF) with the presence of W(CO)_6_ to obtain the PtNi-W/C catalyst [45, 46]. Figure 1b illustrates the schematic nanostructure of PtNi-W/C catalyst. It has been reported in the literature that the preparation of Ni-containing nanoparticles such as Pt_3_Ni [9], PdNi [47] involves the use of weak acidic reducing agents such as AA or BA to achieve the modulation of Ni content, wherein ascorbic acid can provide OH^−^ for the surface oxidized Ni [48, 49] In this process, the Ni species can be released from the surface of the particles, allowing their capture to the carbon black surface, and hence the modification of the surface composition thereafter.

The PtNi-W nanocrystals, showing an average size of ~ 9.6 nm, are uniformly dispersed on the carbon supports (Fig. S1a, b). The nanocrystals display a typical core–shell structure (Fig. 1c, d) in high angle annular dark-field scanning transmission electron microscopic image (HAADF-STEM), which is commonly regarded as a PGM alloy nanostructure with high activity toward the ORR [50]. The 2.3 Å d-spacing corresponds to the lattice spacing of {111} crystallographic planes in face-centered cubic (*fcc*) structure. The energy dispersive spectrometric (EDS) elemental mapping of the single nanocrystal shows that the shell consists mainly of Pt, Ni with W while the core mainly of the Pt and Ni (Fig. 1e–i), indicating that the W atoms exist at the surface region. To further visualize the variation in distribution of difference metal elements, the mapping images are processed in Digital Micrograph software. As shown in Fig. S2, by analyzing the intensity of the signal through a line across the nanoparticle, we can obtain the distribution lengths of Ni at 9.7 nm, Pt at 11.9 nm and W at 10.8 nm. A difference of 2.2 nm between the distribution of Pt and Ni within the same particle suggests a composition gradient across the nanoparticle with Ni mainly distributed in the core and the near surface enriched with Pt. The distribution length of W is obviously larger than that of Ni, indicating that W should also exist in the shell layer. Hence, the nanoparticle features a core–shell structure with a PtNi-based core, as well as Pt- and W-enriched shell layer.

From the HAADF-STEM image at a lower magnification on the carbon particle, scattering bright dots can be found as shown in Fig. 1j, suggesting the existence of scattering metallic species on the carbon support, as single-atom site. The EDS (Fig. 1k–n) reveals that the bright dots consist mainly of W, with very slight amount of Ni atoms. The EDS spectrum recorded at the highlighted region (in Fig. S3a) on the carbon particle, as shown in Fig. S3b can be well assigned to the corresponding species (Fig. S3c, d). It can be clearly seen that no single-atomic Pt species exist on carbon support, wherein the Cu signal comes mainly from the TEM grid. Therefore, the Pt in the PtNi-W nanocrystal are enriched at the surface in the synthetic process, while the W atoms are partially reduced at the surface of the nanocrystal and partially captured at the carbon surface as single-metal-atom sites, as shown in the schematic in Fig. 1b. To further verify the formation mechanism of the core–shell structure, the HRTEM of PtNi/C is shown in Fig. S4a, confirming the highly consistent lattice spacing at the edges and in the core region of the nanoparticles. Additionally, it is evident from the EDS line scanning (Fig. S4b) and elemental mapping (Fig. S4c-f) on a single nanocrystal that Pt and Ni are uniformly distributed throughout the nanocrystal. The control experiment verifies that the W-treatment induces the characteristic core–shell nanostructure. In contrast to the commonly used synthetic strategy of single-atom catalyst mainly by pyrolysis, a low-temperature wet-chemical method is developed in this work achieving the construction of single-atom tungsten sites on the carbon-based surface.

**Fig. 2:**
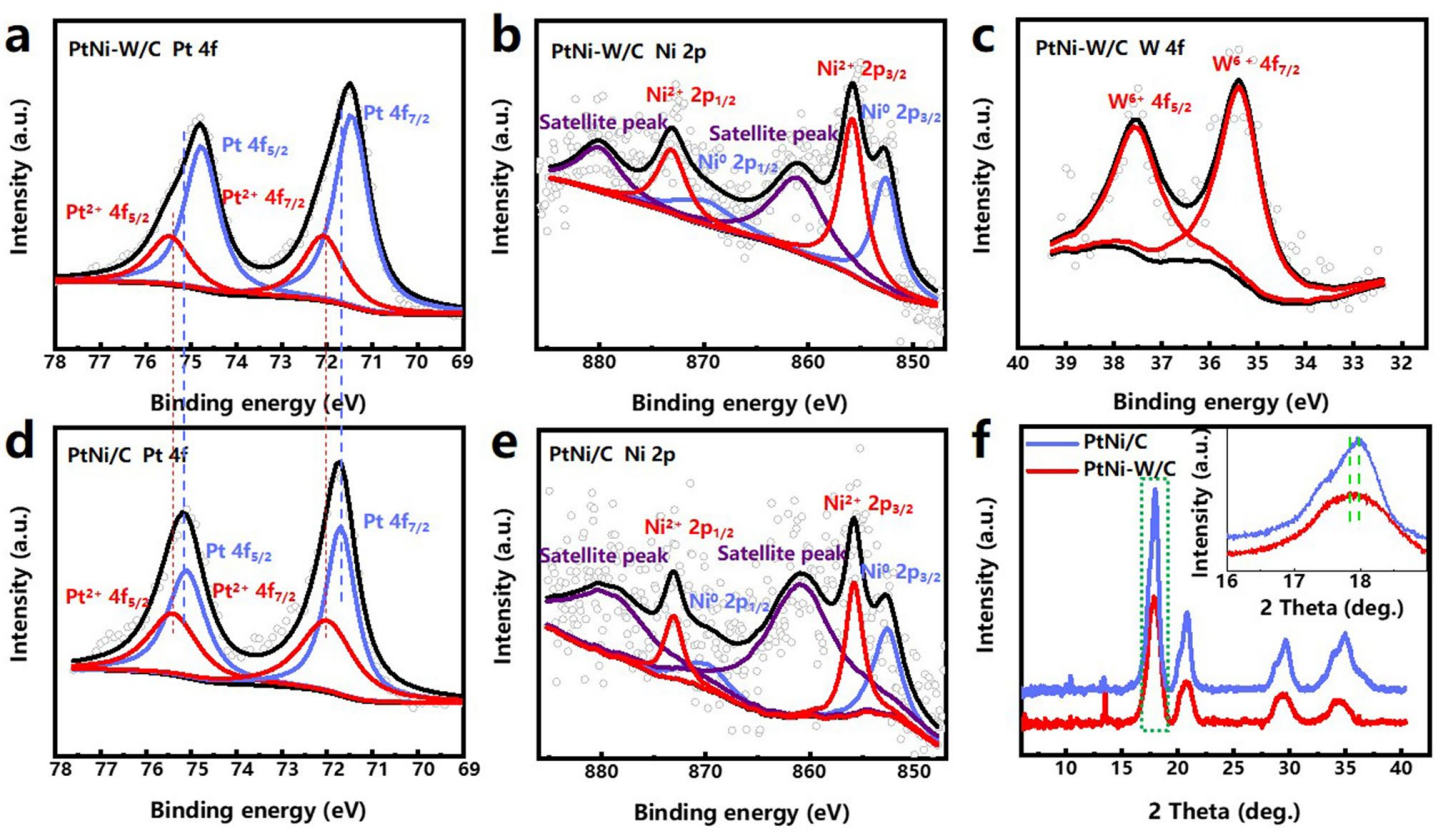
XPS spectra of **a** Pt 4*f*, **b** Ni 2*p*, and **c** W 4*f* core level binding energies for PtNi-W/C and **d** Pt 4*f*, **e** Ni 2*p*, for PtNi/C. **f** Wide-angle X-ray scattering (WAXS) curves of PtNi-W/C and PtNi/C with inset plot of magnified (111) peak of PtNi. The hollow circles indicate the raw data for **a-e**

The chemical states and core level electrons of the key metal elements have been investigated using X-ray photoelectron spectroscopy (XPS). The core level Pt 4*f* spectra suggest that the binding energy of PtNi-W/C is negatively shifted by 0.3 eV compared to that of PtNi/C which serves as comparison sample without W treatment (Fig. 2a, d). It has been reported that the wet-chemical treatment with W(CO)_6_ may involve the reconstruction of the surface atoms for PGM nanoparticles [51]. The core level Ni 2*p* XPS shows that the electronic structure of Ni does not significantly change (Fig. 2b, e). Meanwhile, the W 4*f* XPS exhibits a valence state of + 6 (Fig. 2c), indicating the existence of electronic interactions between W and the neighboring atoms as well as the partial surface oxidation [52]. For the PGM nanocrystals, the transformation from the PtNi alloy to a Pt-rich shell structure could be accompanied by a change in the surface electronic redistribution at the near-surface layer at the presence of the more electronegative W, resulting in a change in the Pt electronic structure and optimization of the d-band center [53, 54]. By comparing the XPS spectra of Pt 4*f*, Ni 2*f* and W 4*f*, it can be inferred that the W atoms could donate electrons to the Pt atoms at the PGM nanocrystal surface. As for the scattering W atoms on the carbon particle surface, considering again the EDS result that confirms the absence of N atoms (Fig. S3d), it can be speculated that the single-atomic W in the oxidation state is dominantly bonded with O, which differs from the N-coordination of single metal atoms by conventional pyrolytic method. The Fourier transform (FT) extended X-ray absorption fine structure (EXAFS) of the W L_3_-edge in PtNi-W/C is shown in Fig. S5. The two main peaks at ∼1.56 and 2.33 Å correspond to the W–O and W-Metal bonds. The main single-atomic W species on the carbon particle can be designated as W_SA_O_3_ in hybrid catalyst, according to the fact that W exhibits a valence state of + 6 along with the W/O ratio (1/3) shown in Fig. S3d. The XPS shows that the Pt/Ni ratio changes from 10/1 to 5/1 based on the surface elements obtained from XPS (Fig. S6). Considering the appearance of Ni single atoms on the carbon black surface, it suggests that Ni atoms migrate from the PGM nanocrystal to the carbon surface in the wet-chemical W treatment probably due to the oxidation by the high-valence W, whereby the surface enrichment of Pt on the PGM nanocrystal is offset by the formation of single-atom Ni, leading to the decreasing Pt/Ni ratio. The discovery that the W makes up to 10% of the surface content could also be correlated to the occurrence of single-atomic W on the carbon surface. A decrease in the Pt content of the bulk (including the weight of carbon support) from 20.2 to 15.3 wt%, as determined by ICP-AES, could be related partially to the inclusion of W with a content of 0.04 wt%, and partially from the detachment of nanocrystals or oxidized leaching of Pt during the W treatment [51].

The wide-angle X-ray scattering (WAXS) patterns of the catalysts are shown in Fig. 2f with an X-ray wavelength of 0.6887 Å, wherein the peaks at 18.10°, 20.9°, 29.75° and 35.10° can be attributed to diffractions of the (111), (200), (220) and (311) crystallographic planes of the face center cubic (*fcc*) structure of the PtNi alloy, corresponding to the peaks at 41.19°, 47.85°, 70.06° and 84.79° when using an X-ray wavelength of 1.5406 Å [55, 56]. In addition, as shown in Fig. S7 with a wavelength of 1.5406 Å, the shifted diffraction peaks in contrast to the Pt confirms that the as-prepared PtNi is alloy phase, while the W treatment further induces the shifts of diffraction peaks to lower 2θ values. Inclusion of W with a larger size creates tensile strain on the shell and resulting in the lower shift of all diffraction peaks. The process can be understood as a thermal treatment in liquid environment, essentially as a surface doping with low content of W. The thermal treatment in the liquid phase could lead to secondary crystallization of the nanoparticles, which improves the crystallinity of the nanoparticles [57]. The inset in Fig. 2f signifies the peak shift of (111), which is related to the global Pt/Ni/W atomic ratio and is also consistent with the core–shell structure of HRTEM [56, 58].

### ORR and Membrane Electrode Assembly Performance

**Fig. 3:**
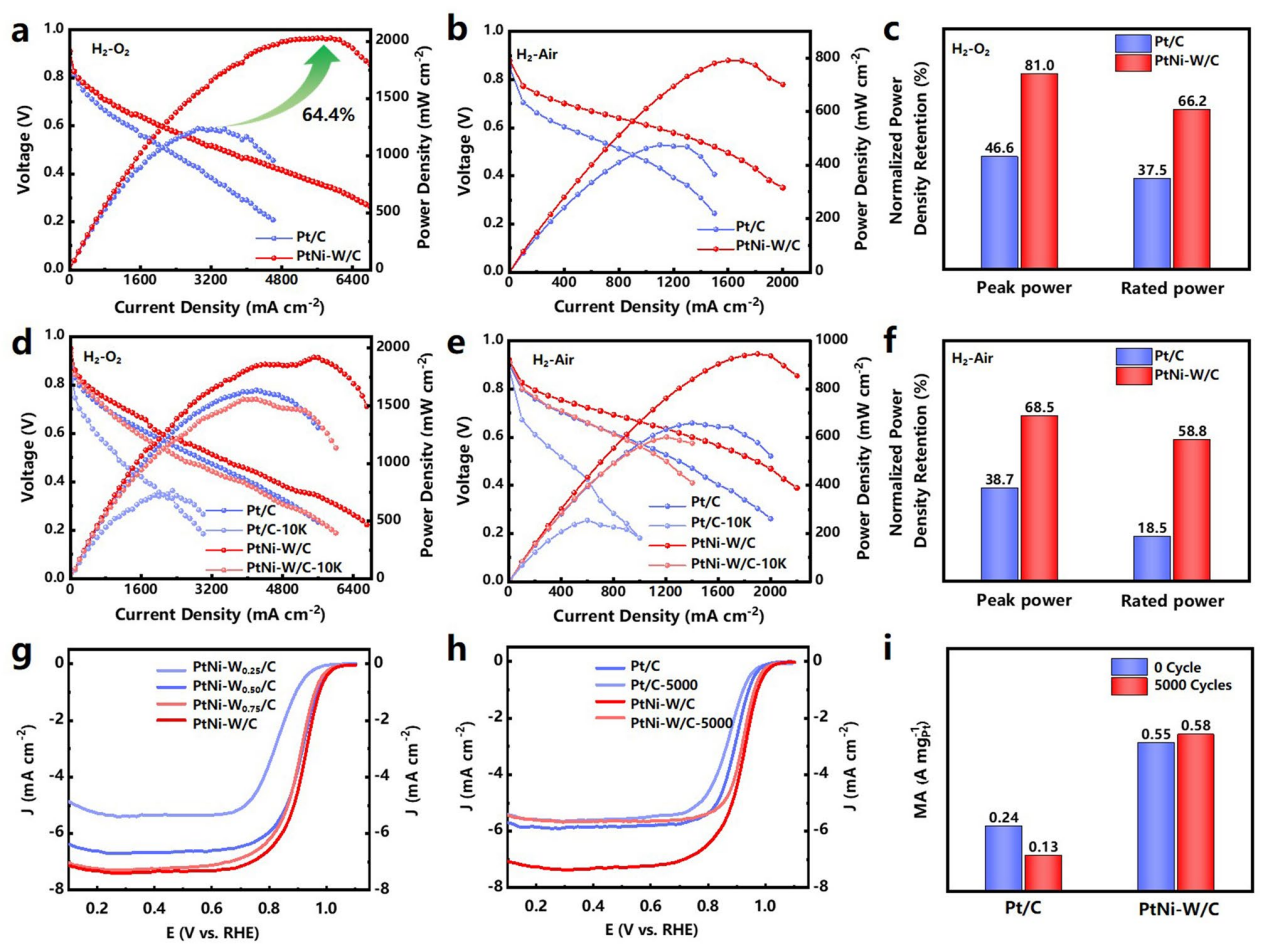
**a** H_2_-O_2_ and **b** H_2_–Air fuel cell polarization (left axis) and power density (right axis) plots with cathode loading of 0.05 mg_Pt_ cm^−2^ for commercial Pt/C (blue spheres) and PtNi-W/C (red spheres). **d** H_2_-O_2_ and **e** H_2_–Air fuel cell polarization and power density plots of commercial Pt/C and PtNi-W/C before cycling and after 10 k potential cycles between 0.6 and 0.95 V with cathode loading of 0.1 mg_Pt_ cm^−2^. **c** H_2_-O_2_ and **f** H_2_–Air degree of power density retention before and after 10 k durability Test. For all the fuel cells polarization tests, the membrane was M820.15, temperature 80 °C, absolute pressure of 1.5 bar for both anode and cathode, relative humidity 100% and anode loading 0.1 mg_Pt_ cm^–2^. **g** LSVs curves of PtNi-W_0.25_-C, PtNi-W_0.5_-C, PtNi-W_0.75_-C and PtNi-W/C.** h** LSVs curves of Pt/C and PtNi-W/C at initial cycle and 5,000th cycle. **i** Summary of mass activities for electrocatalysts before and after 5,000 cyclic potential polarizations

The catalysts are then implemented in single-cell MEA to evaluate their performances as cathode catalysts. We compared the power generation capabilities of PtNi-W/C and commercial Pt/C catalysts using both H_2_-O_2_ and H_2_-Air conditions, to assess the viability of the catalyst. The catalyst ink is prepared by dispersing the calculated amount of catalyst in water/isopropanol mixed solvent with perfluorosulfonic acid ionomer (PFSA, DuPont D2020), and then coated onto the membrane (Gore, M820.15) using air brush. The PtNi-W/C catalyst is coated onto the cathode by controlling the PGM loading an ultra-low level at 0.05 mg_Pt_ cm^−2^, while the anode employs commercial Pt/C catalyst (60 wt%, Johnson Matthey) [18, 59]. The current–voltage (I-V) polarization curves are recorded at 1 bar of completely humidified O_2_/Air to calculate the powder density as shown in Fig. 3. For the MEA used to assess the catalyst stability, a higher cathode loading (0.10 mg_Pt_ cm^−2^) is used.

In the H_2_-O_2_ testing condition (Fig. 3a), the MEA with PtNi-W/C catalyst displays a polarization curve with a peak power density (PPD) of 2030 mW cm^−2^, 64.4% higher as compared to that of the MEA with commercial Pt/C catalyst (1235 mW cm^−2^). At 0.67 V, the MEA with PtNi-W/C generates a current density at 1.2 A cm^−2^, whereas the control MEA can only generate 0.75 A cm^−2^, leading to the rated power density (RPD) of 799 mW cm^−2^ (with PtNi-W/C) compared to 504 mW cm^−2^ (with Pt/C) [60, 61]. It can be clearly seen that the voltage of MEA with PtNi-W/C is significantly higher in the activation polarization control region than that with commercial Pt/C, suggesting a faster reaction kinetics on the PtNi-W/C. It also displays a superior current density at the Ohmic polarization control region, which is related to the lower Ohmic resistance in the PtNi-W/C as evident by EIS measurement (Fig. S8a). The more obvious difference in the concentration polarization region originating from the mass diffusion reveals that the PtNi-W/C facilitates a substantial improvement in mass diffusion. It could be associated with the fact that the catalyst containing W atoms in atomic state could facilitate the transport of protons by forming non-stoichiometric H_x_WO_3_ species which initiate the hydrogen spillover [21-23, 62]. Therefore, in addition to the ionomer network, the mass transfer in the catalyst layer is further enhanced owing to the highly efficient inorganic proton transport network anchored on the carbon surface, and hence significant lowering the overpotential. In H_2_-Air testing condition (Fig. 3b), although the MEA with PtNi-W/C suffers from higher oxygen transport resistance (Figs. S9 and S10), it still shows a PPD 64.4% higher than that with Pt/C. This is associated with the lower Ohmic and reactive resistances suggested by the electrochemical impedance spectroscopy (Fig. S8b), indicating the benefit of the hybrid microstructure. It is worth noting that for the MEAs fabricated with the ultra-low cathode loading at 0.05 mg_Pt_ cm^−2^, they achieve the performance very similar to what has been reported with Co–Pt/C catalyst [63]. It can reach 88% of the highest performance of MEA made by using PtCo/C-based ORR catalysts [64]. Furthermore, under hydrogen–oxygen testing condition, the PtNi-W/C-based MEA exhibits a comparable performance compared with the recently reported MEA with Pt-Ni-based catalyst [65], indicating the catalyst reported in this work has high potential for real application. The recently reported MEA performances based on PGM ORR catalyst is summarized in Table S1.

In accordance with the testing protocol suggested by the US department of energy (DOE) to assess the catalyst stability, accelerated durability test (ADT) is performed with cyclic voltage sweeps between 0.6 and 0.95 V on MEA [61]. Figure 3d displays the *V-I* polarization with power density curves in H_2_-O_2_ condition following the ADT of 10,000 cycles. For the MEA with commercial Pt/C catalyst, the rated power density and peak power density retains only about 46.6% and 37.5% of the beginning of life (BOL) performance, respectively (Fig. 3c), suggesting the fast degradation of MEA with ultra-low PGM loading. The Nyquist Plot of the control MEA (Fig. S8c) shows a semicircle with larger radius compared to the EIS at BOL, which can be ascribed to the larger reaction resistance induced by the structural decay of the catalyst. The low real axis intercept of the Nyquist Plot with has shifted to higher values, meaning an increasing Ohmic resistance, which suggests that the performance decay is dominated by the elevating resistance of the carrier transport inside the cell. However, the MEA using PtNi-W/C cathode demonstrates higher durability (Fig. 3c), with the RPD and PPD retaining 81.0% and 66.2% of its BOL values, respectively. The MEA displays a mitigated degradation both in the Ohmic polarization control region dominated by the cell resistance, and in the concentration polarization control region dominated by the mass transfer. The degradation can be attributed to the increased carrier transport resistance due to the retarded transport of protons originating possibly from the disrupted proton transport network enabled by single-atom W site.

It can be noted that Fig. 3d shows a slight reduction in power density for the MEA with PtNi-W/C with increasing PGM loading compared with the MEA used in Fig. 3a. One may find the fluctuation of the polarization curve at the high current density region (~ 1.6 A cm^−2^), as well as the abnormal power density profile in proximity to the PPD region in Fig. 3d, which could be associated with inefficient mass transfer and water management due to the formation of water droplet in the thicker catalyst layer with higher catalyst loading [66, 67]. Therefore, water management has become a limiting factor in the high current density region. As for the MEA tested with H_2_-Air circumstance (Fig. 3e), the water generated in the reaction does not act as the mass-transfer constraint. With increased loading, the MEA with PtNi-W/C exhibits a higher PPD of 946 mW cm^−2^ in H_2_-Air, although the oxygen gain exists compared with the MEA operated with pure oxygen [68, 69]. The MEA with Pt/C retains only 38.7% and 18.5% in PPD and RPD of BOL, respectively. In contrast, the RPD and PPD of PtNi-W/C can preserve 68.5% and 58.8% of BOL following the durability test under H_2_-Air. The durability test reveals that PtNi-W/C displays higher activity and stability under air condition compared to Pt/C. The EIS results in H_2_-Air (Fig. S8d) show the similar trend to that measured in H_2_-O_2_. We also prepared MEA by using PtNi/C at cathode, as another control MEA to confirm the effect of W on the high activity of PtNi-W/C (Fig. S11). The MEA only displays PPDs of 583 and 275 mW cm^−2^ with oxygen and air, respectively, revealing the essential role of W in the boosted performance in device.

To further verify the intrinsic activity of the catalyst for the electrocatalytic ORR, a rotating disk electrode (RDE) test is carried out at room temperature in a 0.1 M HClO_4_ solution saturated with O_2_. As shown in Fig. 3g–i, PtNi-W/C displays a more positive onset potential and hence a higher current density at 0.9 V than that of Pt/C. The MA (0.55 A mg_Pt_^−1^) calculated using the kinetic current after the mass-transfer correction is higher than that of Pt/C. In addition, we have calculated the turnover frequency of the catalyst according to the method used in the literature [73]. The TOFs of PtNi-W/C is 0.42 e^−^ site^−1^ s^−1^ at 0.9 V (vs. RHE), which is 6 times that of Pt/C. The comparison of the TOF numbers to the literature is listed in Table S2.

A higher diffusion limiting current density, which has been boosted to over 7.5 mA cm^−2^ at the rotating rate of 1,600 rpm, is repeatedly discovered from the PtNi-W/C during the RDE measurement which has been rare for the PGM-based catalysts [70-72]. A series of catalyst samples with various W contents are prepared for the RDE measurement, as can be seen in Fig. 3g. When increasing the W content, the limiting current density is found to be increasing, indicating that the elevated diffusion limiting current could be associated with the existence of above-mentioned W_SA_ site on the carbon surface. However, it seems that the diffusion limiting current reaches a maximum with the W content, possibly due to the saturated proton network channel on carbon black surface with a limited number of single-atomic W sites [74, 75]. Besides, the PtNi/C, as comparison catalyst, is prepared using the same protocol as PtNi-W/C except that the W precursor is not added in the wet-chemical treatment. Meanwhile, the Pt-W/C, also as a comparison, is prepared by using the Pt/C (without Ni) in the wet-chemical treatment with W precursor. In obvious contrast, the diffusion limiting currents of PtNi/C and Pt-W/C are found to fall in 5.5 ~ 6.0 mA cm^−2^, which is normal for PGM-based catalysts (Fig. S12). The above comparison suggests that the formation of single-atomic W site could be associated with the Ni. Considering the finding from STEM results that there are Ni species on the carbon support, which is commonly overlooked by previous research, it is speculated that the formation and stable anchoring of W_SA_ on the carbon surface could be facilitated mainly by the Ni species. Previous research on catalyst synthesis has not adequately highlighted this phenomenon [76, 77]. To see whether the elevated diffusion limiting current is caused by the formation of 3D catalyst coating layer on glass carbon electrode (GCE), the film thicknesses are compared by depositing the same amount of catalysts on GCE. It can be seen that the films formed by the PtNi-W/C and Pt/C catalysts on the GCE do not show obvious difference in thickness, as shown in Fig. S13, which eliminates the effect of the porous structure.

The formation of W_SA_, which leads to the existence of tungstic species on the carbon surface, could be the structural origin of the abnormal diffusion limiting current [78]. The diffusion limiting current is influenced by geometrical factors and the local oxygen/proton concentrations at the electrode, according to the equation of Faradaic current reported by Chen et al*.* [79] as:

9 $${\text{i}} = {\text{zFA}}_{{{\text{ECSA}}}} {\text{k}}\mathop \prod \limits_{{{\text{i}} = 1}}^{{\text{n}}} \left( {{\text{c}}_{{{\text{A}}_{{\text{i}}} }}^{{\text{s}}} } \right)^{{{\text{a}}_{{\text{i}}} }}$$

As for the concentration terms in Eq. (1), many studies normally made a tacit assumption that the current is limited by the diffusion of oxygen, by assuming the surface concentrations of oxygen and proton as zero. However, if the local concentration of proton varies due to the accelerated proton transport, the following Equation deduced from the Levich Equation will not always stand valid [79].

10 $$i_{l} = 0.2\frac{z}{{x_{i} }}A_{{{\text{geo}}}} FD_{{A_{{O_{2} }} }}^{\frac{2}{3}} \nu_{0}^{{ - \frac{1}{6}}} \omega^{\frac{1}{2}} c_{{O_{2} }}^{b}$$

Therefore, the elevated proton supply rate facilitated by the W_SA_, and hence the none-zero surface local concentration of protons, leads to the elevated diffusion limiting current density dominated by oxygen [80, 81]. More specifically, the protons captured by the W_SA_ site, leading to the formation of tungstic acid can be again released and supplied to the catalytic site. As a result, the effective proton concentration of the localized region at the catalytic site is hence increased, resulting in the elevated diffusion limiting current. The half-reaction stability test is also carried out to evaluate the intrinsic stability of the PtNi-W/C catalyst. As displayed in Fig. 3h, compared with Pt/C, both of the ECSA and polarization curves of PtNi-W/C show much lower degradation. In particular, the PtNi-W/C catalyst retains its initial MA with even a slight increase in ECSA, compared with the retention of MA by 54.2% and ECSA by 83.0% for commercial 20wt% Pt/C (Figs. 3i and S14). This is associated to how the mass activity is calculated through the polarization curves. It can be found that, after the stability test, the diffusion limiting current density is lowered back to a normal value, possibly due to the full de-protonation of W_SA_ site that may result in the lowered proton centration, which is consistent with the smooth-decay polarization curves after the fuel cell durability test, whereby the W single atom binds to the carbon substrate with a mitigated interaction compared with the strong bonding in conventional single-atom catalyst obtained by pyrolysis [82-84].

To verify the reason for the performance decay, we further characterized the catalyst taken after the stability test (in MEA) by HAADF-STEM, as shown in Fig. S15. The experimental results show that the nanoparticles partially retain the core–shell structure after the stability test, while the metal species on the carbon black surface are changed. As shown in STEM and mapping images on a single alloy nanoparticle, i.e., Fig. S15a-d, the Pt signal area is still larger than that of Ni, indicating that the Ni still tend to reside in the core of the nanoparticles. The mapping images are processed in Digital Micrograph software. As shown in Fig. S16, we can obtain the distribution lengths of Ni at 8.6 nm and Pt at 9.6 nm. As shown in Fig. S15e, the single-atomic sites still can be found with fairly high density on the carbon black surface. The EDS spectrum (Fig. S15f) on the support (corresponding to the highlighted region in the yellow rectangle) reveals the existences of Pt, which may originate from the dissoluted Pt in the alloy nanoparticles. As also shown in the spectrum, the W species still exist on the carbon black after the stability test. In addition, one may find that there are some other impurity elements on the carbon. Combining the above information, it can be seen that the appearance of impurity elements in the catalyst, possibly originating from the environment as well as the dissoluted metallic species from the alloy nanoparticles, inevitably destroys the proton transport channels constructed by the WO_3_ sites, leading to the full de-protonation of W_SA_ site, and hence the decreased diffusion limiting current density to normal values.

### Analysis of Proton Accessibility and Oxygen Resistance

**Fig. 4:**
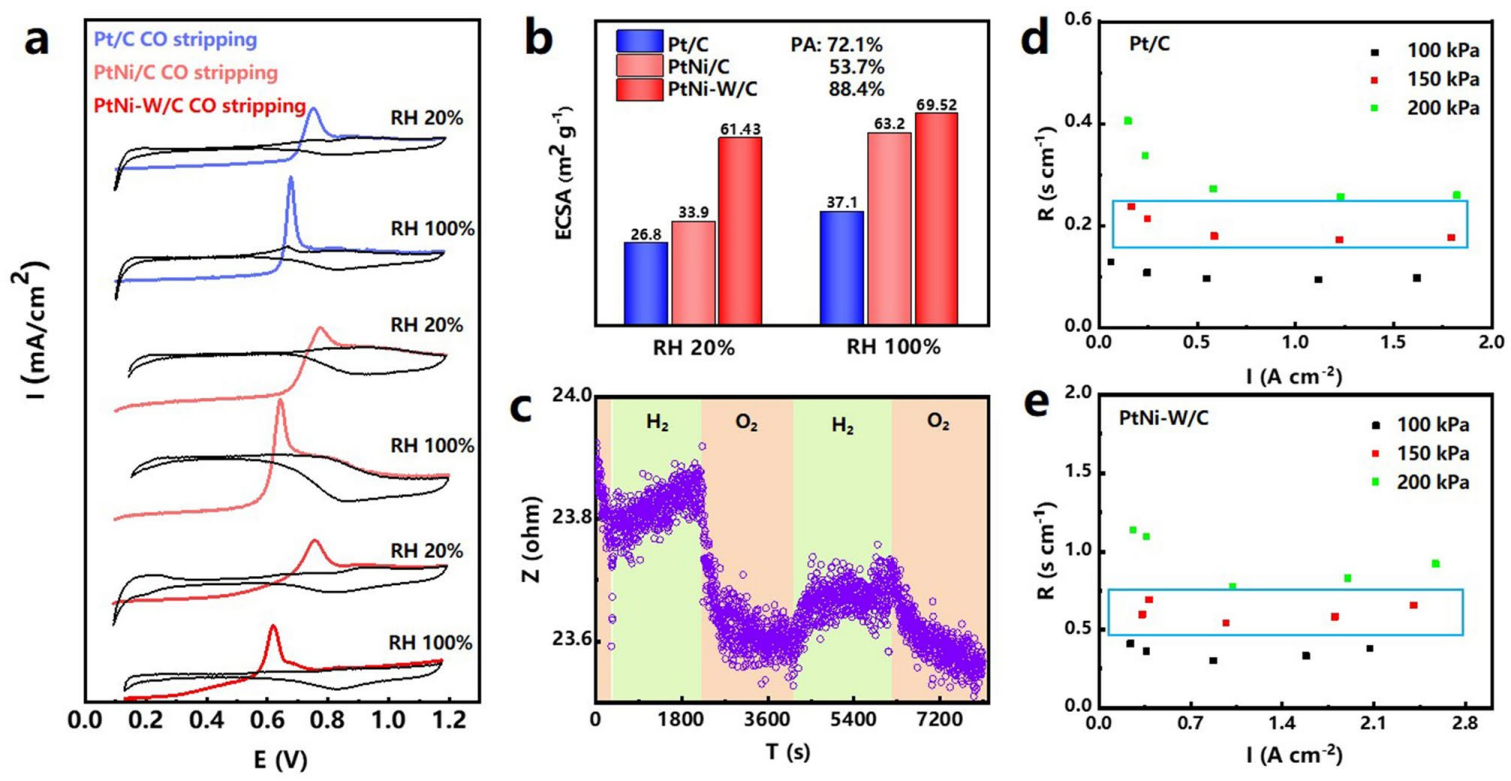
**a** CO stripping curves of commercial Pt/C, PtNi/C and PtNi-W/C at RH 20%, RH 100%. **b** ECSA comparison (proton accessibility) of commercial Pt/C vs. PtNi/C vs. PtNi-W/C. **c** Resistance response of PtNi-W/C under H_2_-O_2_ conditions at room temperature. **d**, **e** Total O_2_ mass transport resistance vs limiting current at 100, 150 and 200 kPa for commercial Pt/C and PtNi-W/C

To validate the hypothesis of the enhanced proton transport in the electrode facilitated by the W species, CO stripping tests in the MEA device are compared at various relative humidities (RH, 20% and 100%) to assess the proton accessibility (PA) in the catalyst layer [19]. The PA, the ratio of ECSAs measured at low and high RH values which involves proton conduction in water, can be used to assess the coverage of the ionomer and its distance of proton to the Pt particles in its proximity in the proton transport network in the catalytic layer. The commercial Pt/C displays a PA of 72.1% based on its ECSA at 20% RH (26.8 m^2^ g^−1^) and at 100% RH (37.1 m^2^ g^−1^), whereas PtNi-W/C presents a PA of 88.4% based on its ECSA at 20% RH (61.4 m^2^ g^−1^) and 100%RH (69.5 m^2^ g^−1^) and PtNi /C presents a PA of 53.7% based on its ECSA at 20% RH (33.9 m^2^ g^−1^) and 100%RH (63.2 m^2^ g^−1^). (Fig. 4a–e), indicating the easy access of protons to the catalytic sites at dry conditions. It is reasonable to assume that the inclusion of W species could facilitate the proton coverage with enhanced transport to the Pt sites. It can be seen that PtNi/C has a lower proton accessibility, and in comparison, the PA of PtNi-W/C is improved by 34.7%. It indicates that the addition of W atoms significantly enhances the proton accessibility of the catalyst. The value of proton accessibility could be influenced by the existence of additional pores in catalyst. To maximize proton accessibility, we use Vulcan XC-72R carbon black rather than porous carbon, which eliminates any additional proton-conductive pores.

It is worth noting that CO is stripped at lower potential on PtNi-W/C surface, which is related to the optimization of adsorption energy of surface oxygen species [86]. The ECSA values determined by the CO stripping, as well as the trend, have clearly deviated from the values obtained from half-electrode measurement (Fig. S14). It is commonly accepted that the ECSA of Pt/C measured in the MEA device is usually lower compared with the value determined on half-electrode setup [87, 88]. However, the PtNi-W/C catalyst layer exhibits higher ECSA value in MEA, which serves to corroborates the existence of a proton network microstructure other than the ionomer binder in the catalytic layer and as the evidence of the effective implementation of the W_SA_. We further performed CO stripping experiments on the RDE to verify the accuracy of the half-cell ECSA, with the curves shown below as Fig. S17. The ECSA_CO_ reached 35.8 m^2^ g^−1^ which is similar to the ECSA_H_ value of 34.2 m^2^ g^−1^, indicating that the measured value of ECSA is accurate. After the polarization test, the PtNi-W/C catalyst inevitably undergoes dissolution of Ni atoms and hence the exposure of more Pt surface considering the size of the PtNi-W/C does not increase as much as the Pt/C that undergoes serious ripening, which could be the reason for the increased ECSA.

In addition, the oxygen resistance is determined as shown in Figs. 4d, e and S8-S9 to investigate the influence of oxygen transport. The total oxygen resistance measured at various oxygen partial pressures reveals that PtNi/C catalyst layer exhibits a substantially higher oxygen diffusion barrier [89]. We can see that the total oxygen transport resistance of PtNi-W/C is substantially lower than that of PtNi/C at a back pressure of 150 kPa. It can also be found in the linear fit in Fig. S10 that with the addition of the W, PtNi-W/C has a lower intercept, i.e., the local oxygen transport resistance is optimized.

Nonetheless, the single-atomic W site in the hybrid catalyst, different from the Pt/C, may increase the binding strength of the ionomer to the catalyst surface, influencing the roughness factor of PEMFC electrodes and thus imposing a larger barrier to oxygen transport [19, 90]. We further carried out the electrochemical tests and MEA tests using less Nafion, e.g., comparing the performances of RDE electrodes and MEAs fabricated by adding 80%, 90% and 100% of the normal amount of Nafion in Fig. S18. The diffusion limiting current densities decline by only very little, still much higher than the normal diffusion current density. The MA values of the catalysts with 80%, 90% and 100% are calculated to be 0.50, 0.52 and 0.55 A mg_Pt_^−1^. Furthermore, in the MEA tests, the MEA with 90% Nafion content displayed a similar cell performance to the normal MEA especially at the activation polarization control region, while the MEA using 80% of normal Nafion content shows a reduced power density at concentration polarization control region (3,500 mA cm^−2^). This result indicates that the proton transport network formed by the single-atomic W can compensate the reduced amount of Nafion and enhance the proton transport.

It has been reported for the bulk tungsten oxide that their interaction with proton could be associated with the metal–insulator transition effect [23]. To better understand the enhanced proton transport facilitated by the structure design of the PtNi-W/C and their correlation to the W_SA_ site, we have also validated the electrical responses of PtNi-W/C catalysts at controlled atmospheres (hydrogen or oxygen). By periodically switching the dissolved gases in the acid electrolyte between hydrogen and oxygen, the electrochemical impedances of the catalyst electrode can be obtained. As shown in Fig. 4c, the electrode presents distinctive electrochemical resistance variation profile. When exposed to oxygen-saturated electrolyte, the protons adsorbed on W_SA_ react with oxygen, whereby the proton transport pathway is opened up, and hence carrier concentration as well as the proton conductivity significantly increases, leading to decreased resistance. The abnormally higher diffusion limiting current can be well explained according to the Ohm's law considering the W site as metallic state. Upon switching into the hydrogen environment, the W_SA_O_3_ combines again with protons, forming a tungstic acid structure (Brønsted acid) which is chemically stable [5]. It blocks the proton transport by lowering the carrier concentration and anchoring the protons, whereby the resistance increases similar to the insulator. Based on the impedance response effect induced by the protonic acid structure in PtNi-W/C catalyst, an extended proton transport network microstructure inside the catalytic layer is hence formed at operation condition, meaning proton transport network does not exist in the catalyst layer with only ionomer. Therefore, we assume that the proton transport network created by single-atom W species on the carbon black, is mostly responsible for the proton accessibility. It can mitigate the strong dependence of three-phase interface microstructure relying on the ionomer. Importantly, the resistance response indicates that the proton acidification process can occur in cyclic manner, which could be beneficial to the practical cell operation.

### DFT Analysis

**Fig. 5:**
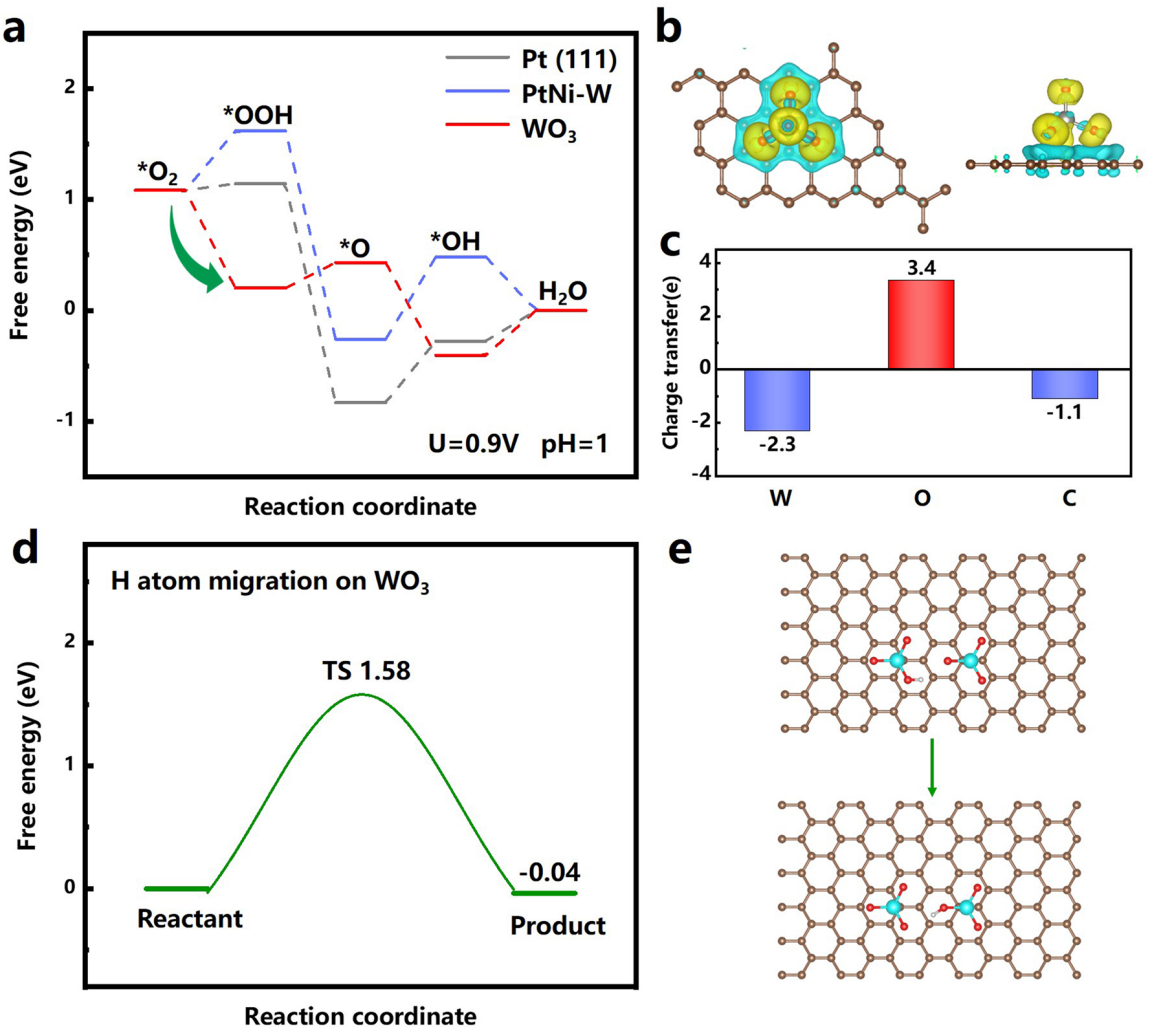
**a** Free energy diagram of the ORR pathways on Pt(111), PtNi-W and WO_3_. **b** Adsorption configuration of O* on W_SA_O_3_ active sites with charge density differences typically (cyan represents holes and yellow represents electrons). **c** Corresponding Bader charge analysis. **d** Energy profiles and **e** calculated models for H atom migration on W_SA_O_3_. (Legend: silver = Pt, green = Ni, blue = W, red = O, white = H, brown = C)

To better understand the enhanced activity of PtNi-W/C catalyst, a DFT calculation is performed. The detailed information on the DFT calculation parameters as well as the selected model (Fig. S19) is provided in the Supplementary Material. The thermodynamic potential barriers on three parallel ORR reaction surfaces are calculated, including the Pt (111), PtNi-W alloy and on W_SA_O_3_, by a combination of multi-step proton and electron reaction sequences (Figs. 5 and S19) [91, 92]. The Pt(111) shows reaction energy diagram consistent with literatures [93, 94]. Calculations reveal that the free energy for the *OOH step at the W_SA_O_3_ site is 1.42 eV lower than that at the PtNi-W site, suggesting a dramatically lowered energy barrier and a favorable *OOH formation, indicating that O_2_ may be quickly activated by W_SA_O_3_ to create *OOH. The green arrow in Fig. 5a shows that the down-hill energy diagram of the *OOH formation step at the W_SA_O_3_ sites. Since the formation of *O seems to be a slight uphill process at the W_SA_O_3_ site but goes very smooth at the Pt site, we can reasonably speculate that the *OOH could transport preferentially to the Pt site on PtNi-W surface in atomical proximity or at the WO_3_/PtNi-W interface to proceed. Combined with the elemental mapping on the carbon surface, the single-atomic W sites exist at fairly high density, indicating the existence of immediately adjacent W_SA_O_3_ sites in the proximity to the alloy nanoparticle surface. The above experimental results indicate that the network formed by the W species and the highly possible hopping of oxygen from W site to the nanoparticle. Some studies have also reported that the adjacent heterogeneous sites also synergistic on the reaction intermediates, e.g., Co–Ni Sites [95], Fe–Mn/N–C [96], etc. Hence, it is possible that the synergistic effect between W_SA_O_3_ and Pt site can be accomplished.

As for the conversion of *O to *OH, the Pt site (on bot pure Pt and PtNi-W) shows difficulty in terms of free energy level, while the W_SA_O_3_ site seems to favor the process. Therefore, it can be seen that the conversion energetics of sub-steps on Pt and W_SA_O_3_ show complementarity, and hence the rate-retarded steps, mainly the formation of OOH* and *OH at the PtNi-W route, can be accelerated due to a cascade catalytic mechanism for the nearby dual sites [97]. To reveal the relationship between W_SA_O_3_ and carbon local coordination structure during the ORR, a typical structure of O* on W_SA_O_3_ active sites is displayed with charge density differences and Bard charge analyses (Fig. 5b, c). Figure 5c shows that during the catalytic processes, more electrons are donated from W and C to O, verifying the strong interaction between W_SA_O_3_ and carbon support [98].

Furthermore, to investigate the proton migration on WO_3_ through DFT [99], a theoretical calculation on the transition state (TS) of the proton transport pathway between the W_SA_O_3_ site is carried out, representing the proton transport from a near-center site to a W_SA_O_3_ site close to the edge (at the alloy nanoparticle position). The calculation shows that the energy of the final state decreases by 0.04 eV after the proton migration, indicating that the proton migration is favored in energy perspective. In addition, we found that the TS energy barrier of protons during migration is also lower than that on bulk WO_3_ which TS is 2.04 eV [99], proving that proton transport on the surface of carbon is theoretically possible, as shown in Fig. 5d, e. Because of facilitating proton spillover effect in the solids [100], the mode of proton transport in this work is slightly different from the diffusion in liquid driven by the concentration gradient. Thus, it can be seen as an additional supply of protons within the solid phase for ORR, whereby the net proton concentration and transport will be increased in the PtNi-W/C catalyst.

## Conclusions

In summary, a hybrid catalyst is designed and synthesized toward the ORR, which is implemented as cathode catalyst in PEMFC. The structural characterizations confirm its microstructure of PGM-based nanocrystals loaded on carbon-based supporting material with surface single-atom-catalyst. The as-designed catalyst, upon the deployment in MEA device as cathode, demonstrates boosted activity and enhanced stability operated in both H_2_-O_2_ and H_2_-Air. The MEA with the PtNi-W/C cathode at ultra-low loading of 0.05 mg_Pt_ cm^−2^ showed a PPD of 2030 mW cm^−2^, 64.4% higher than that with commercial Pt/C catalyst, owing to the more efficient proton transport. The resistivity in the catalyst layer determined by switching between H_2_ and O_2_, along with the investigation on oxygen resistance, corroborates that the integration of single-atomic W enables an efficient proton transport networks on the catalytic surface. Meanwhile, the first-principle DFT calculation reveals a reaction mechanism by taking the merits of 4e oxygen conversion energetics on both PtNi-W and W_SA_O_3_ that promotes the cascade catalysis of ORR enabling a more efficient reaction pathway. With such a novel synthetic design along with the interesting finding of the hybrid structure, the activity and stability the catalyst can be regulated in a more flexible manner, with its working mechanism properly understood. It offers new avenues for the design of high-performance electrocatalyst with ultra-low PGM loading for PEMFCs.

### Supplementary Information

Below is the link to the electronic supplementary material.

Supplementary file1 (PDF 1929 kb)
