# Supplementary material for: PtNi-W/C with Atomically Dispersed Tungsten Sites Toward Boosted ORR in Proton Exchange Membrane Fuel Cell Devices
Source: Nanomicro Lett. 2023 Jun 2;15:143. doi: 10.1007/s40820-023-01102-9 (PMC10236083; doi:10.1007/s40820-023-01102-9)
Supplement: Supplementary file 1 — Supplementary file1 (PDF 1929 kb) [file 40820_2023_1102_MOESM1_ESM.pdf]

Supporting Information for

PtNi-W/C with Atomically Dispersed Tungsten Sites Toward Boosted ORR in Proton Exchange Membrane Fuel Cell Devices

Huawei Wang[1], Jialong Gao[1], Changli Chen[1], Wei Zhao[2], Zihou Zhang[1], Dong Li[1], Ying Chen[1], Chenyue Wang[1], Cheng Zhu[1], Xiaoxing Ke[3, *], Jiajing Pei[4], Juncai Dong[4], Qi Chen[1], Haibo Jin[1], Maorong Chai[2], Yujing Li[1, *]

[1]Beijing Key Laboratory of Construction Tailorable Advanced Functional Materials and Green Applications, School of Materials Science and Engineering, Beijing Institute of Technology, Beijing 100081, P. R. China

[2]State Power Investment Corporation Hydrogen Energy Company, Limited, Beijing 102209, P. R. China

[3]Faculty of Materials and Manufacturing, Beijing University of Technology, Beijing 100124, P. R. China

[4]Beijing Synchrotron Radiation Facility, Institute of High Energy Physics, Chinese Academy of Sciences, Beijing 100049, P. R. China

*Corresponding authors. E-mail: yjli@bit.edu.cn (Yujing Li); kexiaoxing@bjut.edu.cn (Xiaoxing Ke)

Supplementary Figures and Tables

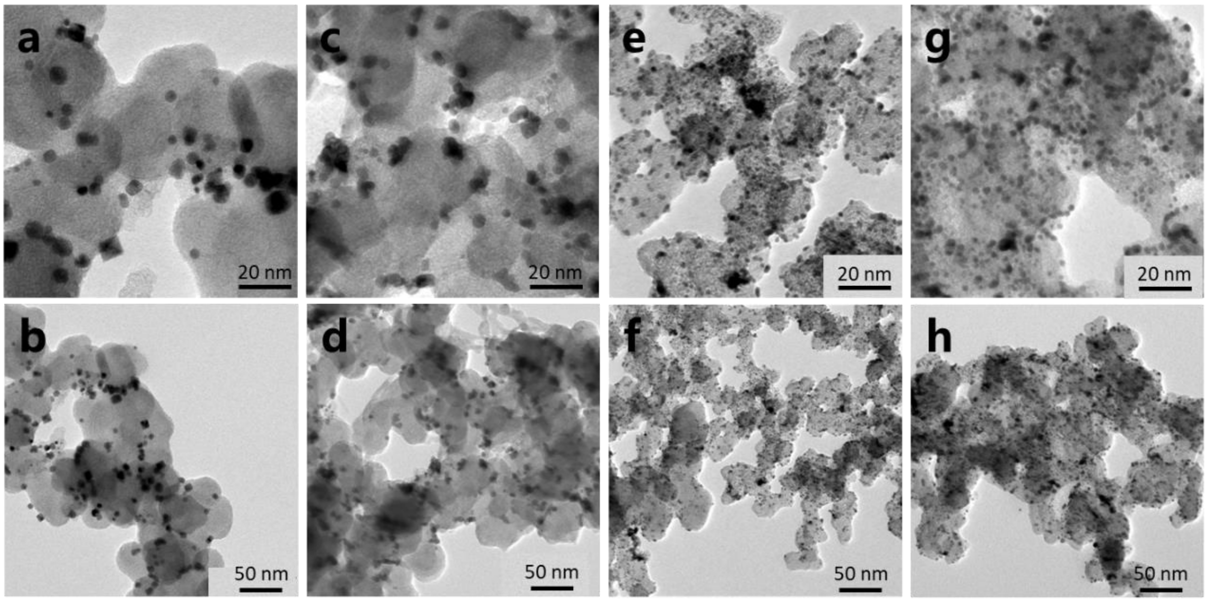

Fig. S1 a, **b** TEM of PtNi-W/C at the beginning and **c**, **d** after 10k durability Test. **e**, **f** TEM of Pt/C at the beginning and **g**, **h** after 10k durability Test

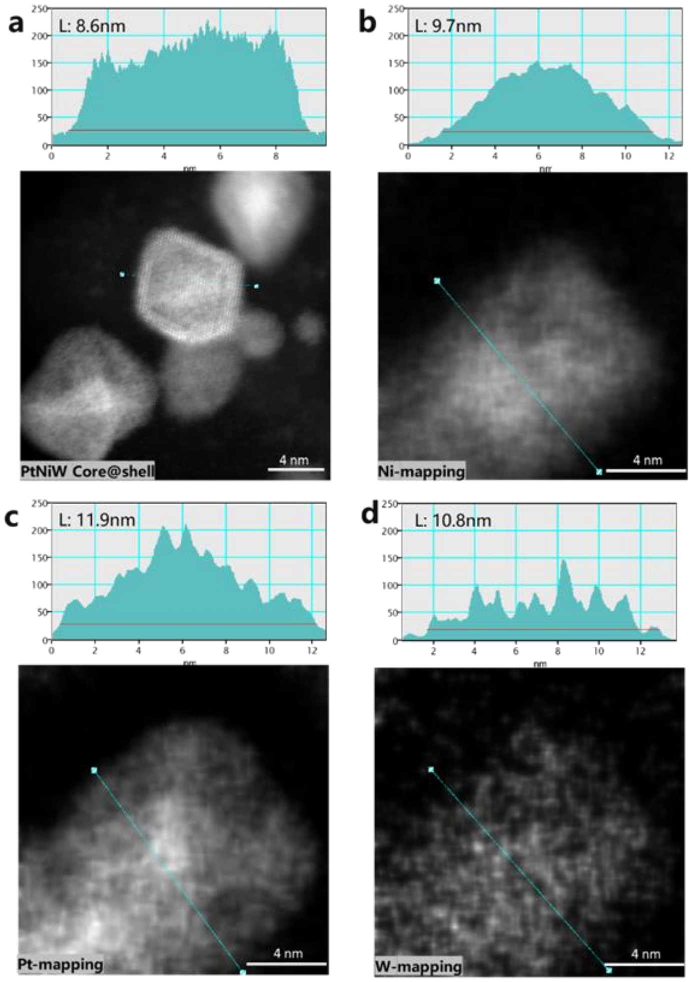

Fig.S2 a Analysis of the elemental mapping for PtNiW nanoparticles, **b** Ni-mapping, **c** Pt-mapping and **d** W-mapping

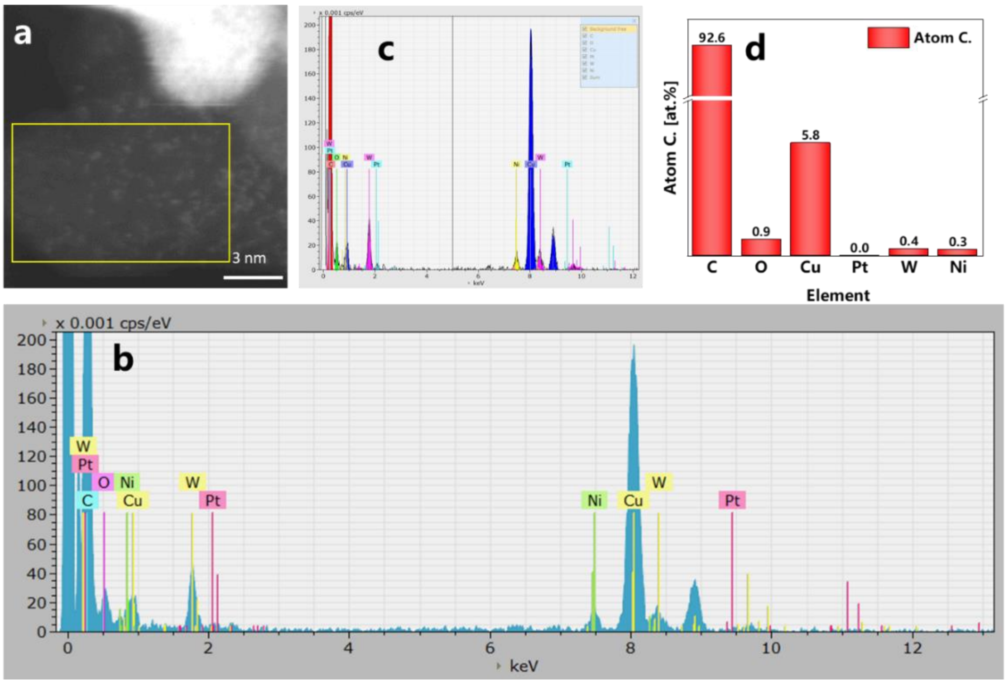

Fig. S3 a HAADF-STEM images of carbon support. **b** EDS for the selected area in the carbon support. **c** Deconvolution calculation of elemental content in the EDS. **d** Elemental content on the support

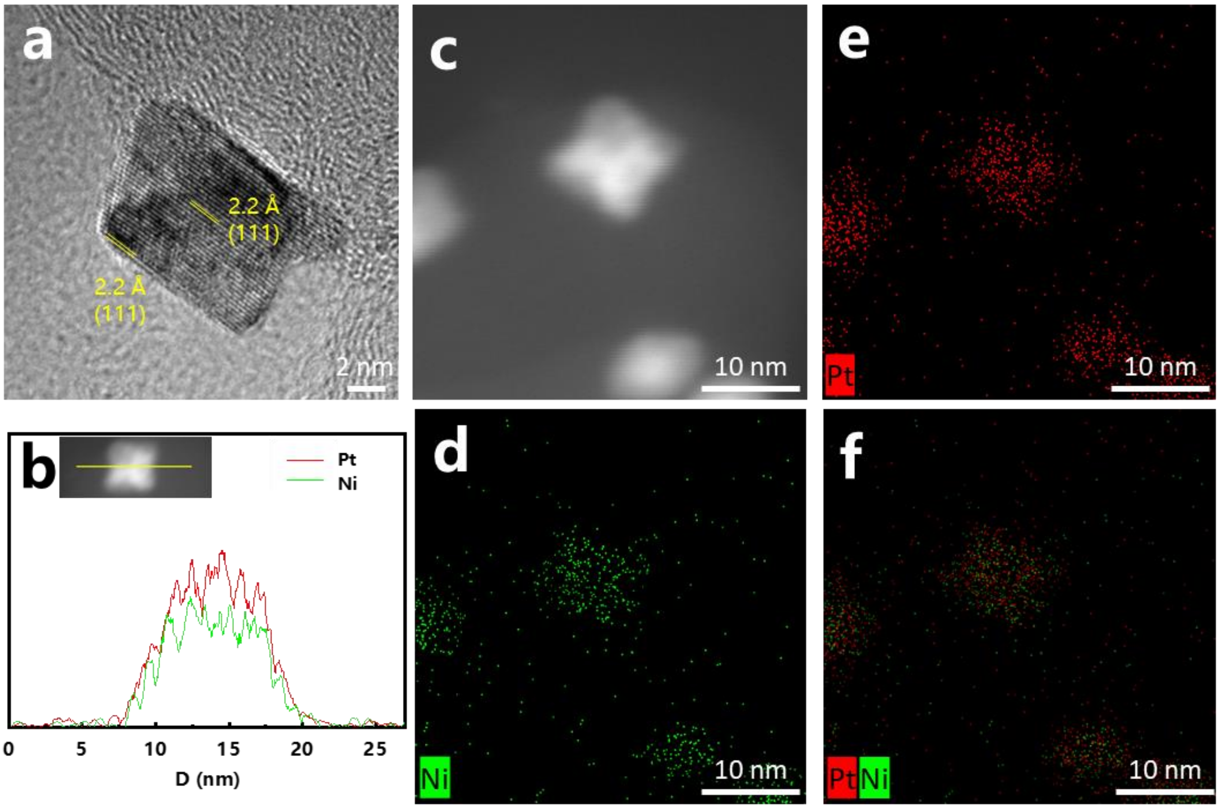

Fig. S4 **a** HRTEM images of PtNi/C. **b** The corresponding EDS elemental line scan along the yellow line in the inset plot. **c** HAADF-STEM images of PtNi/C catalyst. **d-f** Elemental mapping of PtNi nanoparticle, where the Pt, Ni distributions are displayed

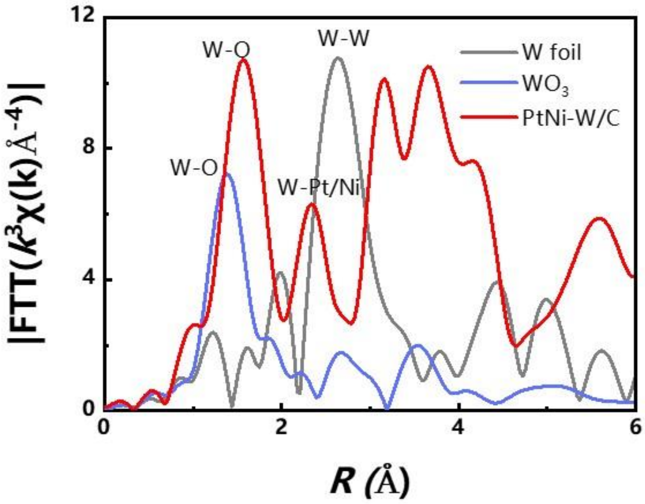

Fig. S5 W L_3-edge EXAFS analysis of PtNi-W/C, WO_3 and W foil in R spaces

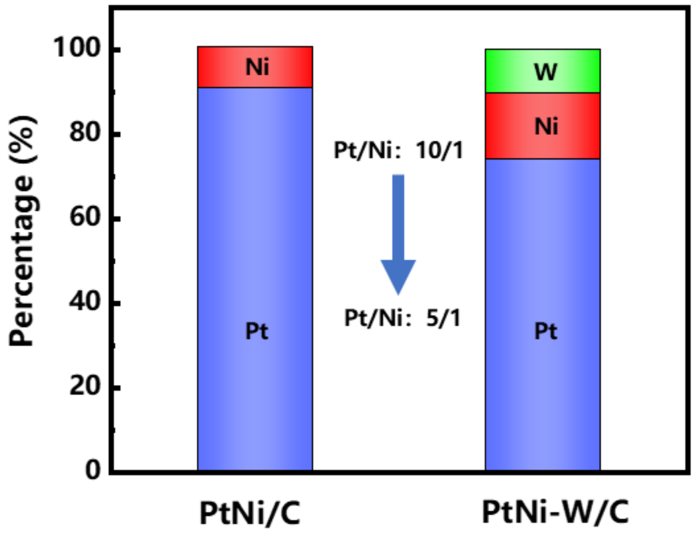

Fig. S6 Ratios of surface elements resulting from XPS

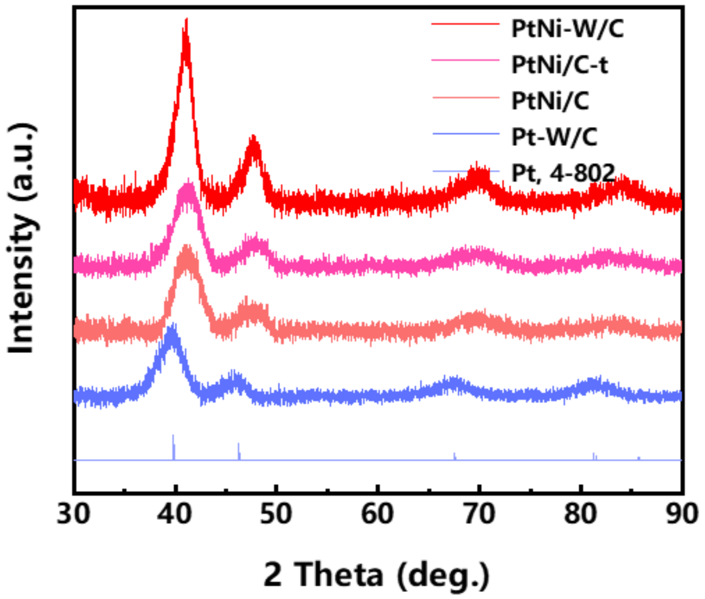

Fig. S7 XRD pattern of Pt (4-802), Pt-W/C, PtNi/C, PtNi/C-t (holding-treatment PtNi/C), and PtNi-W/C

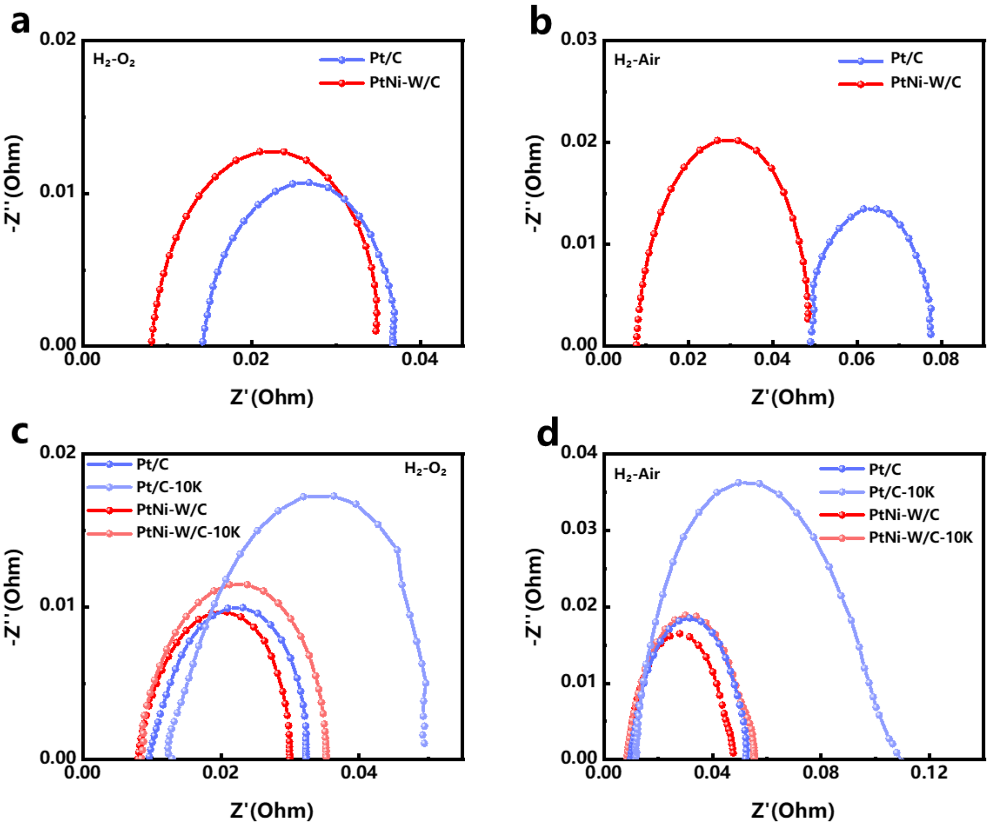

Fig. S8 a H_2-O_2 and **b** H_2–Air EIS with cathode loading of 0.05 mg_{Pt} cm^{-2} for commercial Pt/C (blue spheres) and PtNi-W/C (red spheres). **c** H_2-O_2 and **d** H_2–Air EIS of commercial Pt/C and PtNi-W/C before cycling and after 10 k potential cycles between 0.6 and 0.95 V with cathode loading of 0.1 mg_{Pt} cm^{-2}

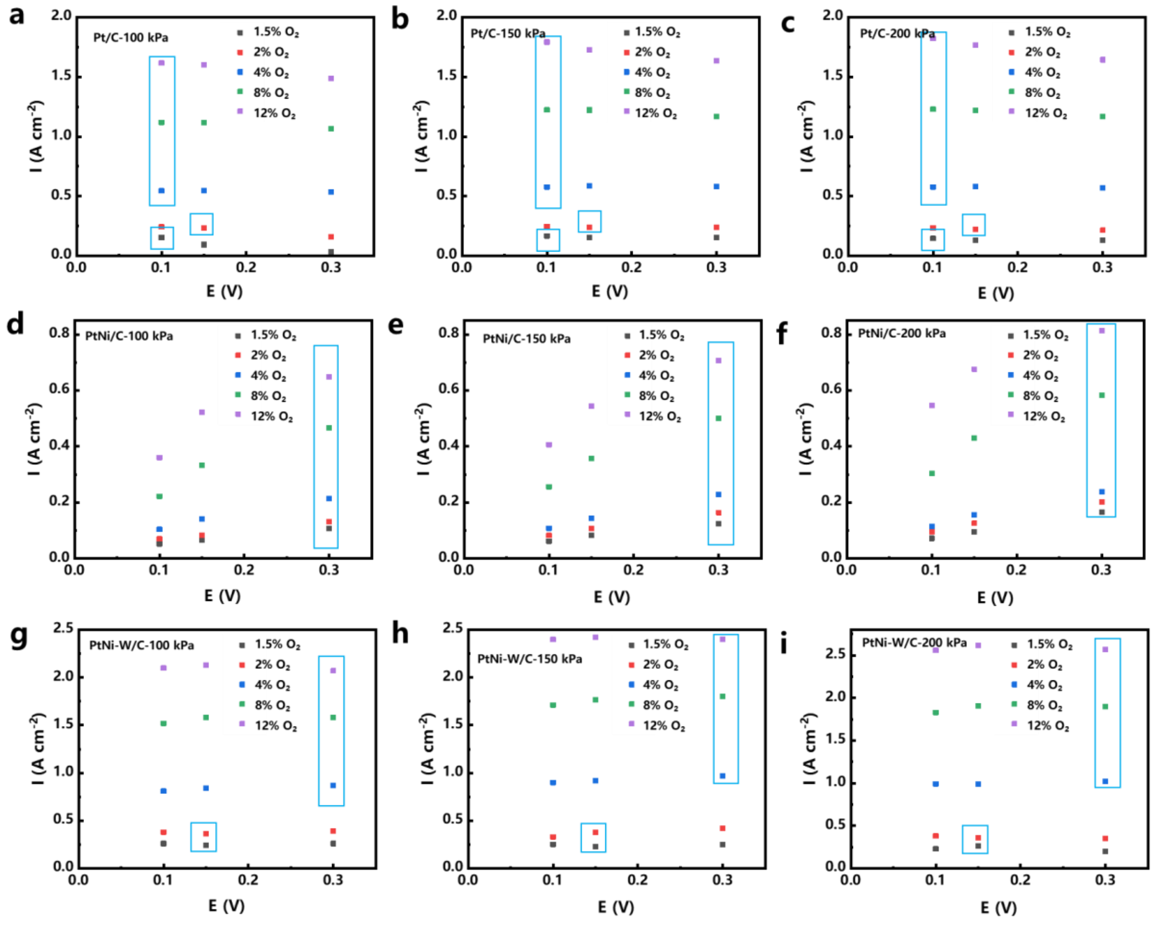

Fig. S9 a-c Limiting current measurement in the potential range between 0.1 and 0.3 V at O_2 concentration of 1.5%, 2%, 4%, 8% and 12% at 100, 150, 200 kPa for commercial Pt/C. Marking the onset of hydrogen evolution. **d-f** Limiting current measurement in the potential range between 0.1 and 0.3 V at O_2 concentration of 1.5%, 2%, 4%, 8% and 12% at 100, 150, 200 kPa for PtNi /C. **g-i** Limiting current measurement in the potential range between 0.1 and 0.3 V at O_2 concentration of 1.5%, 2%, 4%, 8% and 12% at 100, 150, 200 kPa for PtNi-W/C

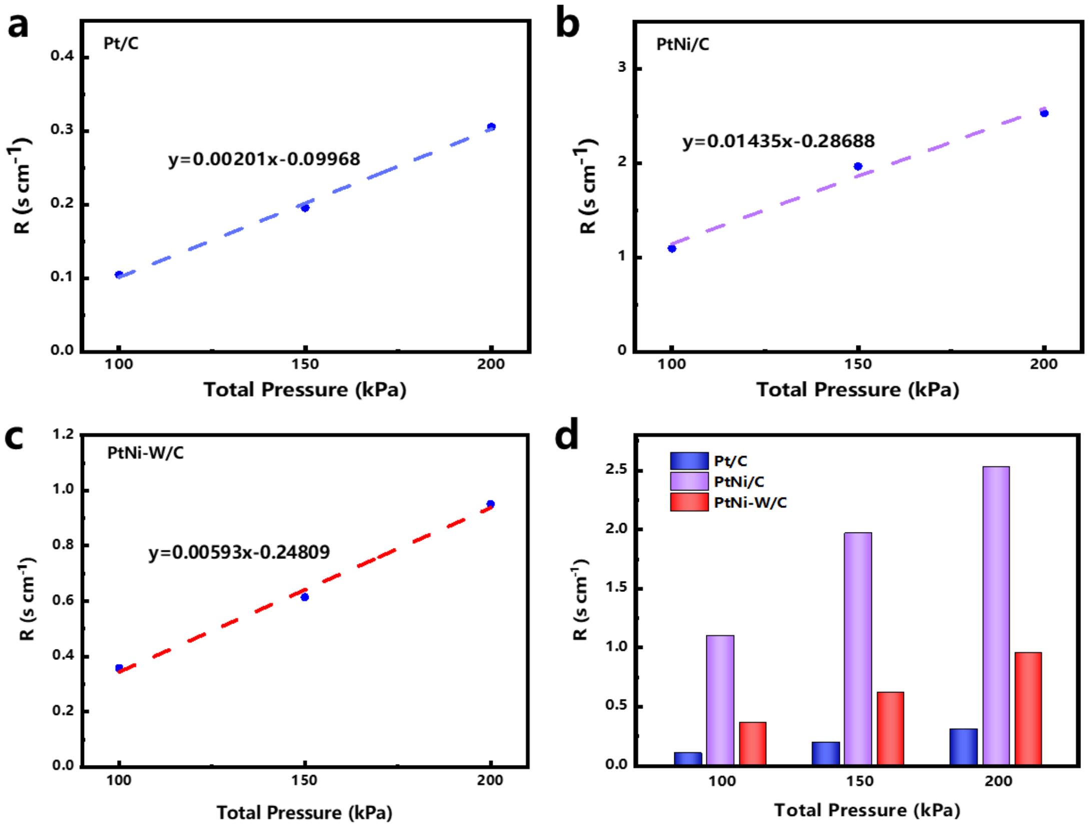

Fig. S10 **a** Total O_2 mass transport resistance for Pt/C, **b** PtNi/C and **c** PtNi-W/C over all O_2 concentrations and the respective pressure. **d** Comparison of oxygen transfer resistance under different back pressure conditions

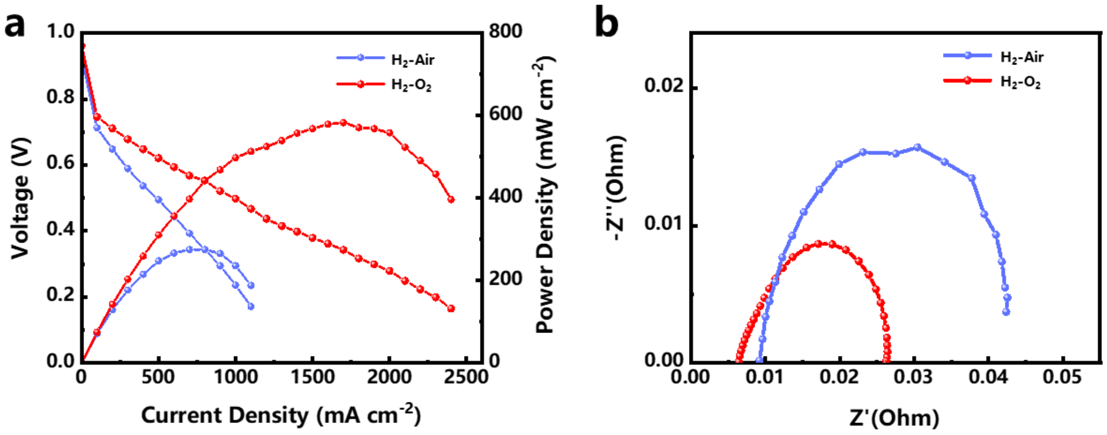

Fig. S11 **a** H_2-O_2 (red spheres) and H_2–Air (blue spheres) fuel cell polarization (left axis) and power density (right axis) plots and **b** EIS with cathode loading of 0.05 mg_{Pt} cm^{-2} for PtNi/C

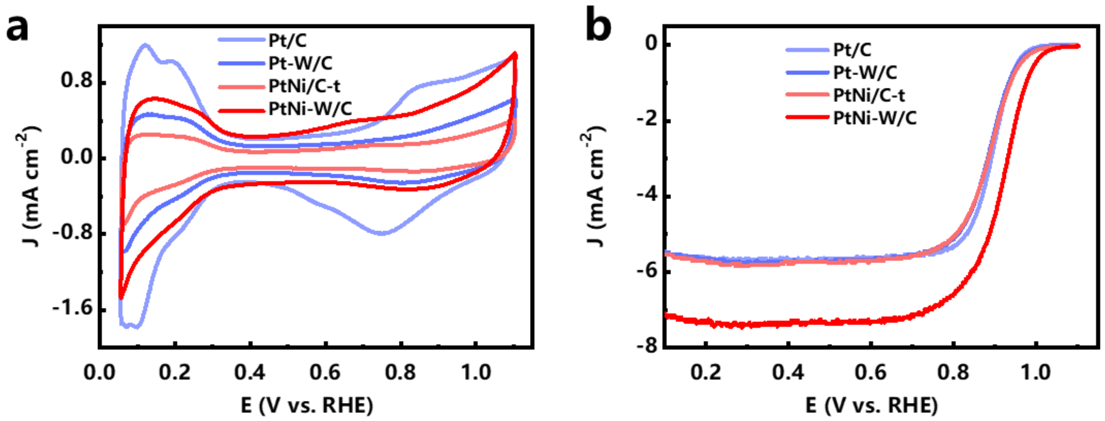

Fig. S12 a CVs and **b** LSVs curves of Pt/C, Pt-W-C, PtNi/C-t and PtNi-W/C

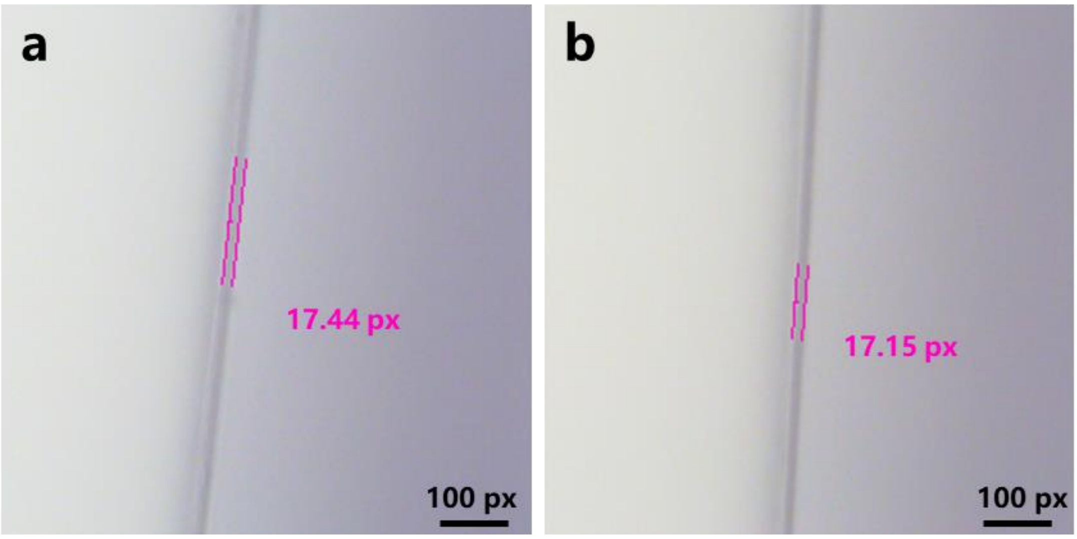

Fig. S13 The thickness of RDE catalyst films formed from **a** Pt/C and **b** PtNi-W/C was compared under optical microscope

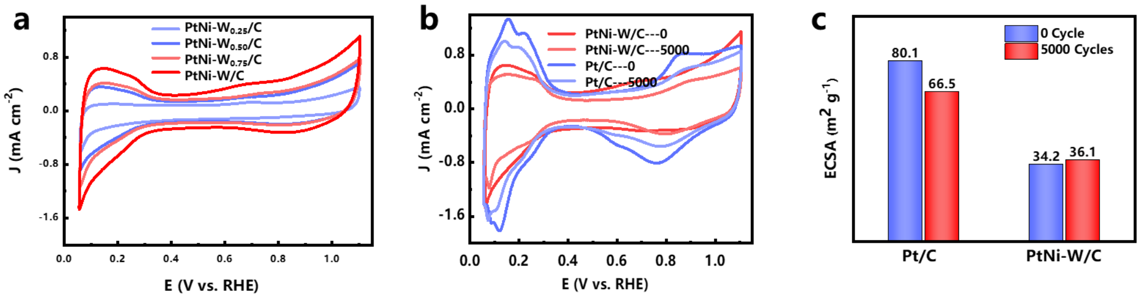

Fig. S14 a CVs curves of PtNi-W$_{0.25}$-C, PtNi-W$_{0.5}$-C, PtNi-W$_{0.75}$-C and PtNi-W/C. **b** CVs curves of Pt/C and PtNi-W/C at 0 and 5,000th CV. **c** Summary of ECSA for electrocatalysts before and after 5,000 cyclic potential polarizations

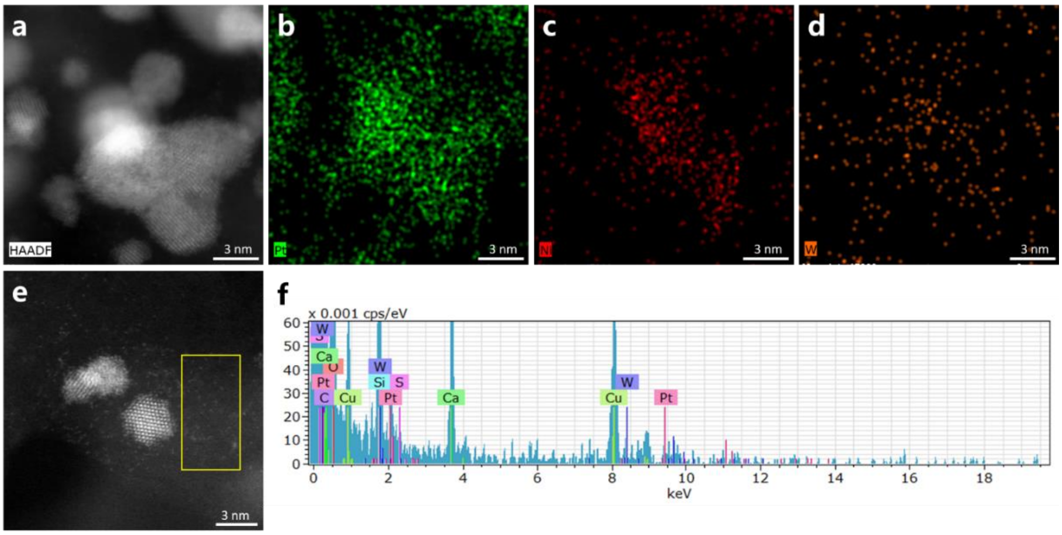

Fig. S15 a-d HAADF-STEM images with elemental mapping of PtNi-W nanoparticle after the stability test (in MEA), where the Pt, Ni, W, distributions are displayed. **e** HAADF-STEM images of PtNi-W/C and **f** EDS for the selected area in the carbon support

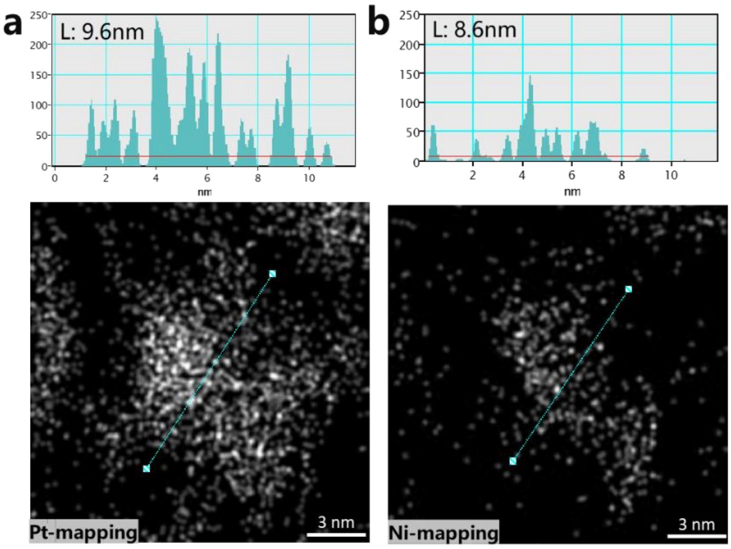

Fig. S16 a Analysis of the elemental mapping for Pt and **b** Ni on PtNiW nanoparticles after the stability test (in MEA)

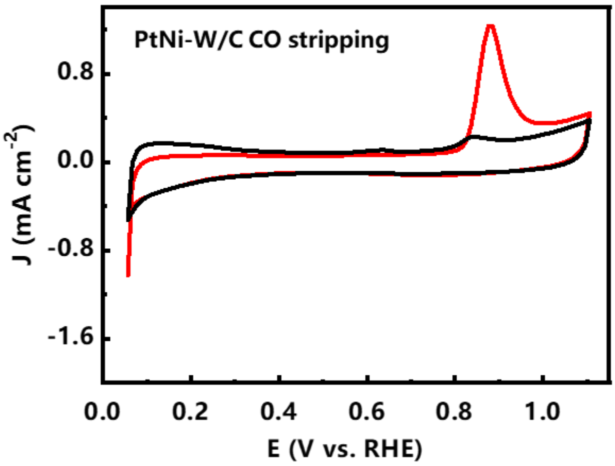

Fig. S17 CO stripping curves of PtNi-W/C at half-cell setup

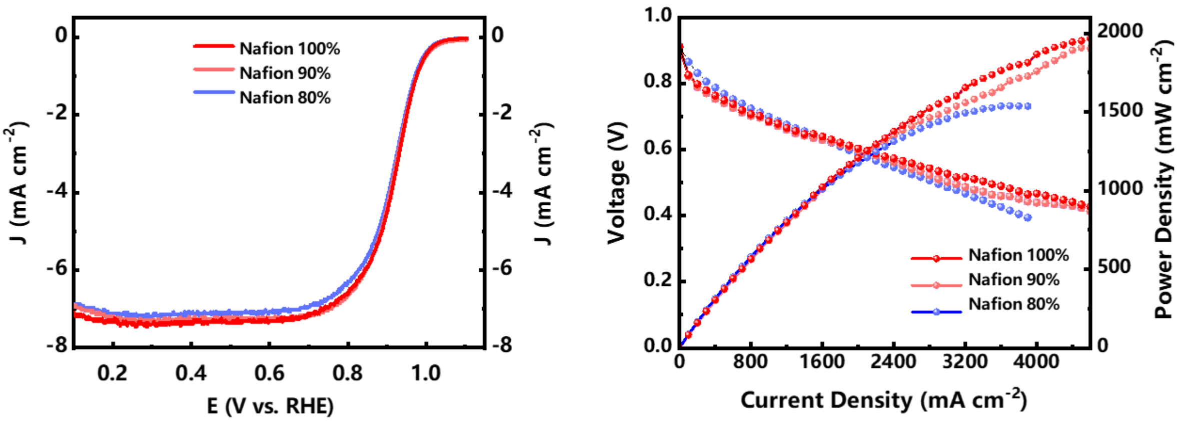

Fig. S18 a LSVs curves of PtNi-W/C using less Nafion. **b** H_2-O_2 fuel cell polarization and power density plots with cathode loading of 0.05 mg_{Pt} cm^{-2} for PtNi-W/C using less Nafion

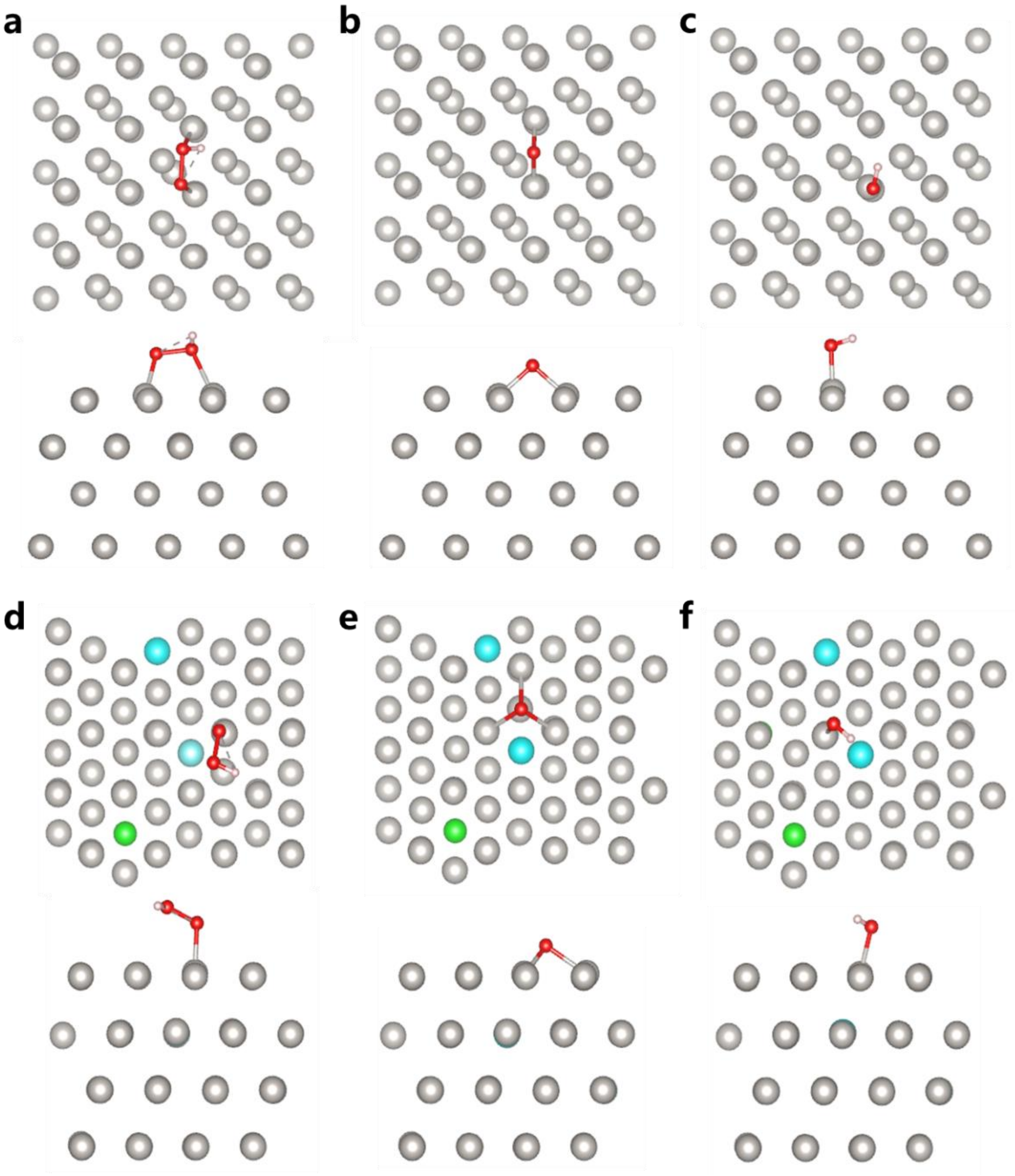

Fig. S19 a ORR mechanism goes through the following intermediates on Pt (111) active sites: OOH*, **b** O*, **c** OH* and **d** OOH*, **e** O*, **f** OH* on PtNi-W (111) active sites (Legend: silver=Pt, green = Ni, blue = W, red = O, white = H, brown = C)

Table S1 The recently reported MEA performances based on PGM ORR catalyst

	Loading (mg_{Pt} cm^{-2})	Peak power density (O_2) (W cm^{-2})	Peak power density (Air) (W cm^{-2})	Refs.
Pt/N-KB 600℃	0.11	/	1.39	[S1]
PtCo i-NPs	0.02	/	1.08	[S2]
Coplanar Pt/C NMs	0.1	1.21	0.55	[S3]
Pt-Fe-N-C	0.015	1.08	0.55	[S4]
Pt-Ni UHT	0.066	0.79	/	[S5]
Pt_3FeCo NSs/C	0.1	1.80	0.88	[S6]
i-CoPt@Pt/KB	0.1	/	1.27	[S7]
Pt_3Co-0.87	0.08	2.25	0.96	[S8]
PtNi-W/C	0.05	2.03	0.79	This work

Table S2 The comparison of the TOF numbers to the literature

	TOF@0.90V	TOF@0.85V	TOF@0.80V	Refs.
commercial Pt/C	/	0.24	0.46	[S9]
ALD-Pt/CNT	0.42	/	/	[S10]
Fe–N/C	/	/	1.6±0.2	[S11]
Fe/N/C	/	/	0.33	[S12]
Ce/Fe-NCNW	0.30	/	/	[S13]
Meso/Micro-FeNSC	/	0.69	/	[S14]
PANI-CM	/	/	1.20	[S15]
PtNi-W/C	0.42	1.10	2.27	This work

Supplementary References

[S1] S. Ott, A. Orfanidi, H. Schmies, B. Anke, H. N. Nong et al., Ionomer distribution control in porous carbon-supported catalyst layers for high-power and low pt-loaded proton exchange membrane fuel cells. Nat. Mater. **19**(1), 77-85 (2020). https://doi.org/10.1038/s41563-019-0487-0

[S2] C.-L. Yang, L.-N. Wang, P. Yin, J. Liu, M.-X. Chen et al., Sulfur-anchoring synthesis of platinum intermetallic nanoparticle catalysts for fuel cells. Science **374**(6566), 459-464 (2021). https://doi.org/doi:10.1126/science.abj9980

[S3] Y. Hu, M. Zhu, X. Luo, G. Wu, T. Chao et al., Coplanar Pt/C nanomeshes with ultrastable oxygen reduction performance in fuel cells. Angew. Chem. **133**(12), 6607-6612 (2021). https://doi.org/10.1002/ange.202014857

[S4] F. Xiao, Q. Wang, G.-L. Xu, X. Qin, I. Hwang et al., Atomically dispersed pt and fe sites and Pt–Fe nanoparticles for durable proton exchange membrane fuel cells. Nat. Catal. **5**(6), 503-512 (2022). https://doi.org/10.1038/s41929-022-00796-1

[S5] J. Liu, S. Liu, F. Yan, Z. Wen, W. Chen et al., Ultrathin nanotube structure for mass-efficient and durable oxygen reduction reaction catalysts in pem fuel cells. J. Am. Chem. Soc. **144**(41), 19106-19114 (2022). https://doi.org/10.1021/jacs.2c08361

[S6] L. Bu, J. Liang, F. Ning, J. Huang, B. Huang et al., Low-coordination trimetallic ptfeco nanosaws for practical fuel cells. Adv. Mater. **35**(11), 2208672 (2023). https://doi.org/10.1002/adma.202208672

[S7] T. Y. Yoo, J. Lee, S. Kim, M. Her, S.-Y. Kim et al., Scalable production of an intermetallic pt–co electrocatalyst for high-power proton-exchange-membrane fuel cells. Energy Environ. Sci. **16**(3), 1146-1154 (2023). https://doi.org/10.1039/d2ee04211h

[S8] X. Feng, Y. Bai, W. Zhang, F. Wang, D. Sun et al., Preparation of sub-1 nm pt3co nanoclusters via a seed-densification strategy for enhanced O2 capture in low-pt-loading fuel cells. ACS Energy Lett. **8**, 628-636 (2022). https://doi.org/10.1021/acsenergylett.2c02579

[S9] L. Cui, L. Cui, Z. Li, J. Zhang, H. Wang et al., A copper single-atom catalyst towards efficient and durable oxygen reduction for fuel cells. Journal of Materials Chemistry A. **7**(28), 16690-16695 (2019). https://doi.org/10.1039/C9TA03518D

[S10] J. Gan, J. Zhang, B. Zhang, W. Chen, D. Niu et al., Active sites engineering of pt/cnt oxygen reduction catalysts by atomic layer deposition. J. Energy Chem. **45**, 59-66 (2020). https://doi.org/10.1016/j.jechem.2019.09.024

[S11] D. Malko, A. Kucernak, T. Lopes, In situ electrochemical quantification of active sites in Fe–N/C non-precious metal catalysts. Nat. Commun. **7**(1), 13285 (2016). https://doi.org/10.1038/ncomms13285

[S12] U. I. Kramm, I. Herrmann-Geppert, J. Behrends, K. Lips, S. Fiechte et al., On an easy way to prepare metal–nitrogen doped carbon with exclusive presence of MEN4-type sites active for the orr. J. Am. Chem. Soc. **138**(2), 635-640 (2016). https://doi.org/10.1021/jacs.5b11015

[S13] J.-C. Li, S. Maurya, Y. S. Kim, T. Li, L. Wang et al., Stabilizing single-atom iron electrocatalysts for oxygen reduction via ceria confining and trapping. ACS Catal. **10**(4), 2452-2458 (2020). https://doi.org/10.1021/acscatal.9b04621

[S14] X. Tang, Y. Wei, W. Zhai, Y. Wu, T. Hu et al., Carbon nanocage with maximum utilization of atomically dispersed iron as efficient oxygen electroreduction nanoreactor. Adv. Mater. **35**(5), 2208942 (2023). https://doi.org/10.1002/adma.202208942

[S15] N. D. Leonard, S. Wagner, F. Luo, J. Steinberg, W. Ju et al., Deconvolution of utilization, site density, and turnover frequency of fe–nitrogen–carbon oxygen reduction reaction catalysts prepared with secondary n-precursors. ACS Catal. **8**(3), 1640-1647 (2018). https://doi.org/10.1021/acscatal.7b02897
